# Application of sliding mode variable structure control algorithm in PMSM vector control system in complex environment

**DOI:** 10.1371/journal.pone.0308417

**Published:** 2024-09-13

**Authors:** Haoran Cui

**Affiliations:** College of Information Science and Engineering, Northeastern University, Shenyang, China; Imperial College London, UNITED KINGDOM OF GREAT BRITAIN AND NORTHERN IRELAND

## Abstract

In order to meet the increasing demand of high-performance control in industrial production, a new sliding mode variable structure control algorithm, Asymptotic Sliding Mode Control (ASMC), is designed in this study to solve the serious chattering problem of sliding mode control. Firstly, a traditional sliding mode exponential approximation law control model and a state space and control function are constructed based on sliding mode control. Secondly, by eliminating the jitter factor, ASMC algorithm is combined with sliding mode control to achieve precise control of permanent magnet synchronous motor (PMSM) and improve its performance. The experimental results indicated that in the simulation experiment, the research system tended to stabilize within 0.2–0.3 seconds, and the system chattering was significantly suppressed. And its output was smoother, the jitter amplitude was significantly reduced by 1/3, and the output torque was more stable. In addition, when the parameter *H*_0_ changed to 2*H*_0_, the overall speed curve did not change much, with only a slight overshoot. The overshoot was only 2.8%, and the change amplitude was maintained at around 25r/min, indicating that the research system had strong self stability performance. In actual experiments, the current command oscillation of the research system was significantly reduced. The local graph showed that the output fluctuation amplitude of the asymptotic approach law actual control was significantly smaller under no-load disturbance. When the *H*_0_ changed towards 2*H*_0_, the actual adjustment time was about 0.1 seconds, which was consistent with the simulation experiment. Therefore, the contribution of the research is that the ASMC algorithm can suppress the chattering problem of the system and improve the approaching speed, thus improving the speed regulation quality of the system. This new algorithm has great theoretical and practical significance for improving the performance of PMSM, and is practical in the actual vector control system of PMSM.

## 1. Introduction

As the speed growth of human society, there is an increasing demand for energy around the world. Among them, electricity is the most common and commonly used energy source, and motors are the core equipment for converting electrical energy to mechanical energy. Their efficiency is directly related to the smooth progress of national energy conservation and emission reduction work [1, 2]. Permanent magnet synchronous motors (PMSM), as an efficient and high-performance motor system, has become the core equipment for converting electric energy into mechanical energy, which has many advantages such as simple structure, small volume, light weight and high efficiency. In addition, in response to the challenges faced by traditional control methods, PMSM also has the advantages of higher dynamic response speed, wider speed regulation range and higher power factor. With the emergence of new high-performance permanent magnet materials, the working efficiency and power density of PMSM have been greatly improved. Therefore, PMSM is an ideal high-efficiency motor system [3, 4]. With the increasing maturity of hardware drivers, the high-performance control strategy of software has severely restricted the further development of high-performance technology and products in China’s PMSM, while foreign technological monopolies and blockades in this field have seriously constrained the development of this field. Therefore, studying efficient PMSM control methods has significant theoretical and practical significance for improving the performance of PMSM [5]. At present, the methods for PMSM vector control mainly include Proportional Integral (PI) control and sliding mode control (SMC). However, the traditional PI control has been unable to meet the growing demand for high-performance control in industrial production. Although SMC has high dynamic response speed and good stability, it often has chattering problems in practical applications. Therefore, it is an urgent task to propose a new control algorithm to overcome these limitations. A new sliding mode variable structure control algorithm, asymptotic sliding mode control(ASMC),is designed based on the asymptotic approach law (AAL), using the approach law as the starting point. Its purpose is to improve the speed control quality of the system while suppressing the system chattering problem, and provide theoretical suggestions for the application and development of PMSM vector control systems. Compared with the existing methods, ASMC algorithm has obvious innovations, which will provide important theoretical suggestions for the further development of PMSM vector control.

The research is composed of four parts in total. The first part is a summary and discussion of the important speed control strategies in the current vector control system of PMSM. The second part is an analysis of the ASMC algorithm combined with AAL. The third part analyzes the performance of ASMC and its application in PMSM control systems. The fourth part is a summary of the entire article.

## 2. Related works

The PMSM speed control system often serves as an important execution link within the system, and its control performance will have a significant impact on the reliability and safety of the entire system [6]. At present, the basic control strategies for PMSM speed control systems mainly include constant voltage frequency ratio control and direct torque control [7]. Li X et al. proposed a high-performance predictive control strategy based on the observer to address issues such as parameter mismatch in the predictive current controller of PMSM speed control systems. This method can deal with the problems such as parameter mismatch, thus effectively improving the robustness of speed control. However, it may introduce a large computational burden and require higher hardware [8]. Zhu L et al. designed a non-linear ADRC, which improved the traditional ADRC by using non-linear function, improved the anti-disturbance performance of the speed control system, and solved the problem of poor robustness of the speed control system. However, this method is sensitive to the selection of non-linear function, and it needs to model the system more accurately. [9]. Li S et al. proposed a control strategy that includes fast response and high-precision stable switching based on adaptive trajectory planning to meet the demands of high-precision stability in PMSM speed control systems, effectively enhancing the stability of the closed-loop system and eliminating overall disturbances. However, this method needs complicated control algorithm and parameter adjustment process, and it is difficult to implement [10]. Dai S et al. proposed the latest compensation scheme for control delay based on constant voltage frequency ratio control to address the issue of improving the performance of PMSM speed control systems. This ensured control performance and stability under high-speed motion. At the same time, it also increased the complexity of the system, which requires high time delay [11]. Wei J et al. combined constant voltage frequency ratio control with direct torque strategy to effectively reduce torque ripple on the basis of wide bus voltage ratio for the control problem of electric vehicle PMSM system, thereby optimizing the control of PMSM [12].

In addition, the optimized direct torque control strategy of Rehman A U et al. reduced the computational burden and improvedits robustness through observer optimization. However, the observer design and parameter adjustment are relatively complex, which may bring difficulties in implementation [13]. Mohammed S A Q et al. improved the direct torque control strategy by utilizing iterative learning to address the issues related to torque ripple in PMSM control systems, effectively accelerating the smooth response while significantly reducing torque ripple. However, this method may require higher hardware and parameter adjustment of the system [14]. Cho D H et al. proposed sensor-less direct torque control for PMSM speed control systems in low-speed regions by utilizing square wave nail magnetic linkage, which eliminates low-pass filters and reduces time delay. However, the overall resource consumption of this technology is high, and the calculation process is relatively complex, which needs further optimization and improvement in the later stage [15]. Elsherbiny H et al. proposed an adaptive fuzzy logic controller based on a detailed analysis of direct torque control and model predictive direct torque technology for the built-in PMSM torque control technology of electric vehicles, effectively strengthening the control of the speed control system. This technology is easily affected by parameters, the calculation process is relatively complex, and further optimization is needed in the later stage [16]. Brosch A et al. integrated direct torque technology and harmonic reference generator into the model speed prediction system control framework to address the issues related to torque and power conversion of PMSM in high-speed operation, effectively improving the performance of the system in both smooth and steady states. However, the system relies too heavily on state parameters and has certain experiments in actual control, which require further optimization in the later stage [17]. The technical characteristics of relevant literature are shown in Table 1.

**Table 1:** List of technical characteristics of relevant literature.

Objective	Method	Advantages	Disadvantages	References
Addressing the issue of mismatched speed control parameters for PMSM	Proposed a high-performance predictive control strategy based on observer	This method can handle problems such as parameter mismatch and has excellent robustness	It may bring significant computational burden and require higher hardware	Li X et al. [8]
Addressing the shortcomings of traditional self disturbance rejection controllers	A nonlinear self disturbance rejection control method has been proposed	This technology has strong anti-interference performance and high system robustness	This method is sensitive to the selection of nonlinear functions	Zhu L et al. [9]
In order to improve the stability and control accuracy of the permanent magnet synchronous motor speed control system	A trajectory planning control strategy adapted to it has been proposed	This technology enhances the stability of closed-loop systems and eliminates overall disturbances	This method requires complex control algorithms and parameter adjustment processes	Li S et al. [10]
Speed regulation issues for PMSM	Adopting a constant voltage ratio control delay compensation scheme	This technology has good stability and control effect	This technology adds complexity to the system and has high latency	Dai S et al. [11]
Control issues related to permanent magnet synchronous motor systems for electric vehicles	Proposed a fusion strategy to optimize system control problems	This technology has good stability and control effect	This technology has poor adaptability and requires regular maintenance	Wei J et al. [12]
To optimize the effectiveness of direct torque control	A direct torque control strategy has been proposed to reduce computational complexity	This technology reduces the computational burden on the system and improves its robustness	The design and parameter adjustment of this technology observer are relatively complex	Rehman A U et al. [13]
To optimize the vibration problem of permanent magnet synchronous motor control system	Proposed an improved direct torque control strategy	This method may require high hardware and system parameter adjustments	The control process is relatively complex	Mohammed S A Q et al. [14]
To improve the effectiveness of sensorless direct torque control	A speed control system for PMSM in low-speed regions has been proposed	The system eliminates low-pass filters and reduces time delay	High cost and complex calculation	Cho D H et al. [15]
In order to improve the torque control effect of automotive MSM	Proposed an adaptive fuzzy logic controller	This technology enhances the control of the speed control system	High computational difficulty	Elsherbiny H et al. [16]
To solve the high-speed energy consumption problem of PMSM	Proposed an integrated model speed prediction system	This system solves control and power conversion problems, improving system stability	The system still has a high latency	Brosch A et al. [17]

According to the research of domestic and foreign scholars on motor control technology, conventional constant voltage frequency ratio control has lower accuracy and does not have good dynamic adjustment performance. Moreover, proportional integral derivative (PID) control technology relies on motor control parameters and is easily affected by environmental and parameter changes. Therefore, the study combines SSMC with vector control and innovatively applies an improved sliding mode variable structure control algorithm to PMSM vector control systems, providing technical support for industrial intelligent manufacturing.

## 3. ASMC algorithm combining asymptotic approach law in PMSM vector control system

This section has two processes. The first process is to model the control of the traditional sliding mode exponential approximation method (EAL). Firstly, the construction of the sliding membrane structure control model includes state space, vector switching function, and control function; Secondly, based on the three control conditions of sliding film control, the sliding film control rate of permanent magnet synchronous motor was effectively derived, and the speed control of permanent magnet synchronous motor based on exponential convergence law was obtained. The second process is to optimize the traditional EAL jitter problem and construct a control model for a new sliding mode exponential convergence law.

### 3.1. PMSM speed control based on traditional sliding mode Exponential Approach Law (EAL)

In response to the increasing demand for high-performance control in industrial production that traditional PID control cannot meet, a new sliding mode variable structure control algorithm was designed based on the asymptotic approaching law, with the approach law as the starting point. In the design of the sliding mode structural control model, the "structure" in the sliding mode variable structure control algorithm does not describe a real physical structure or diagram, but rather qualitatively describes the essential properties of a control system and the overall geometric structure of a system’s state trajectory in the state space [18, 19]. Among them, the state space, vector switching function, and control function expression under the mathematical definition of sliding mode control are shown in Eq (1).


$$\{\begin{array}{c} \dot{P}=f(P,V,\tau ),Q=g(P)\\ Z(P)={\left[z_{1}(P)z_{2}(P)\cdots z_{k}(P)\right]}^{T}\\ v_{i}=v_{i}^{+}(P),z_{i}(P)>0\;or\;v_{i}=v_{i}^{-}(P),z_{i}(P)<0 \end{array}$$ (1)

**Fig 1:**
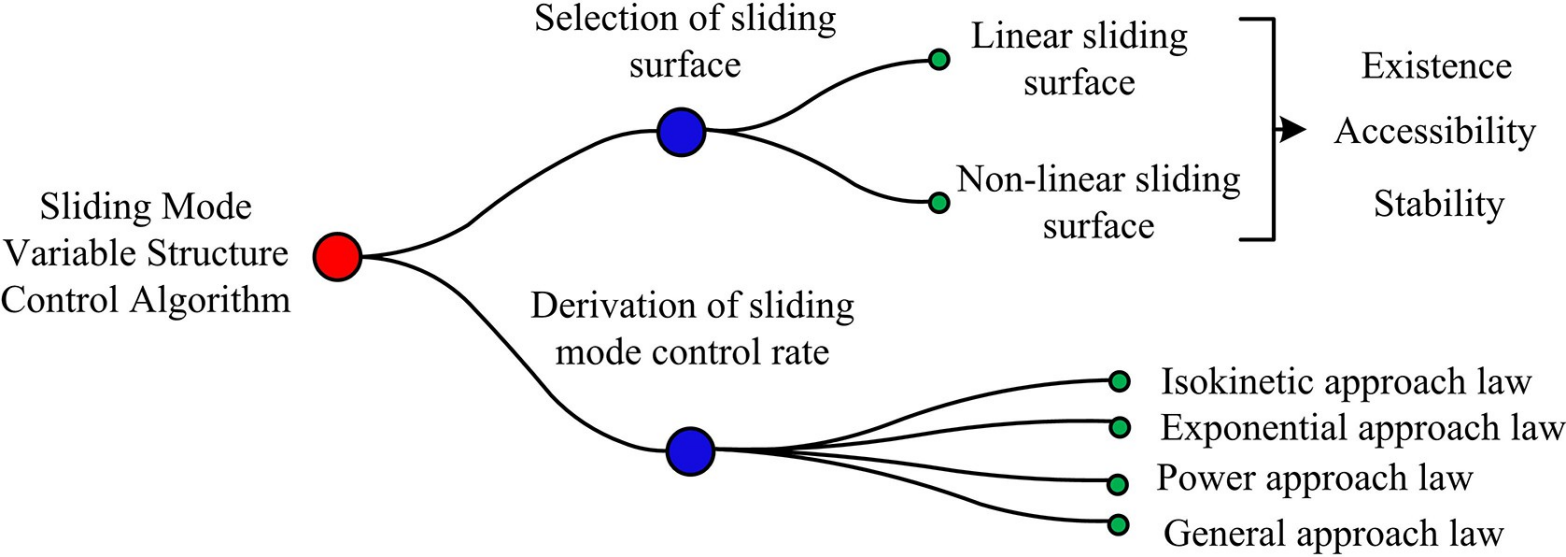
Schematic diagram of sliding mode variable structure control algorithm design content.

In Eq (1), the first row represents the state space of any non-linear system, the second row represents the vector switching function that needs to be determined to have the same dimension as the control input, and the third row represents the control function. Among them, *P* represents the multi-dimensional state vector of the system, and *p* is the internal element of the vector. *V* represents the multi-dimensional control input vector of the system. *v* is its internal element. *τ* represents time. *Q* represents the multi-dimensional output vector of the system. *Z*(*P*) represents the vector switching function expression. *i* represents a dimension, with a maximum value of *k*. By using the control function in Eq (1), any initial point in the system outside the switching plane of the sliding mode surface of *Z*(*P*) = 0 and inside the state space can reach the switching surface within a limited time, and ultimately slide along the switching surface in a relatively stable state. Meeting this condition can be called a sliding mode variable structure control system. On this basis, if the actual state equation of the system is known, the sliding mode variable structure control algorithm can be designed, as shown in Fig 1.

From Fig 1, the design content of the sliding mode variable structure control algorithm mainly includes the selection of sliding mode surfaces and the derivation of SMC rates. The former allows the sliding mode variable structure control system to meet specific performance indicators on the sliding mode surface, while the latter allows any initial point within the state space to reach the switching surface and stably enter the sliding mode within a limited time. Among them, the selection of sliding surface needs to meet the requirements of existence, accessibility, and stability. In the PMSM of synovial structures, there are mainly linear and nonlinear synovial surfaces, and their selection criteria must meet the requirements of controllability, cost, and maintenance. In the above standard research, linear sliding surface is mainly chosen for research, mainly because most motors currently use linear sliding surface control, which has high control accuracy and low control error. The nonlinear sliding surface is similar to PID control, but its calculation is complex and enhances control costs. The linear sliding surface is expressed as Eq (2).


$$Z(P)=B^{T}P,b=[b_{1}b_{2}\cdots 1]$$ (2)

In Eq (2), *B* represents the sliding mode coefficient, and *b* is its internal element. Regardless of which sliding mode is used, the ultimate goal is to gradually converge the system’s state to an equilibrium point under a given control law. The actual state of the sliding mode variable structure control system will eventually approach zero, and the actual approach is exponential. The actual approach speed is adjusted by the sliding mode surface parameter *d*. Although there are various forms of sliding mode surfaces, any form of sliding mode surface must meet the three problems of SMC, namely existence, accessibility, and stability. The existence of sliding mode refers to the presence of a non-zero dimensional region on each side, and each point in this region can reach the surface of the sliding mode within a certain period of time. Sliding mode accessibility refers to the ability of the system to move to the sliding mode surface within a limited time when the *P*(0) is at any position in the state space. Sliding mode stability includes the Lyapunov first method and the second method. The first method determines the stability of the system based on the properties of the solution, and the second method constructs a positive definite Lyapunov energy function from an energy perspective, linking the sign characteristics of the first order differential with energy decay, thereby directly determining the stability of the system. The mathematical expressions or judgment expressions for the three problems are shown in Eq (3).


$$\{\begin{array}{c} \underset{z\to 0^{+}}{\lim}\dot{Z}\leq 0\;and\;\underset{z\to 0^{-}}{\lim}\dot{Z}\geq 0\\ z\dot{z}\leq 0\\ U=\frac{1}{2}z^{2}\Rightarrow \dot{U}=z\dot{z} \end{array}$$ (3)

Lines 1 to 3 in Eq (3) represent mathematical expressions related to existence, accessibility, and stability, respectively. Among them, *U* represents the Lyapunov scalar function expression, where *Z*(*P*) in one row of inequality must pass through the origin and be differentiable in the first order. Under the existence of sliding mode, all points on it can reach the sliding mode surface in a finite time. Compared to existence, accessibility has a wider range of accessibility. For time-varying nonlinear systems, their stability is mainly described by the Lyapunov energy function. Next, it is necessary to effectively derive the synovial control rate for PMSM. The derivation of SMC law is closely related to the motion quality of SMC system. The actual motion of sliding mode variable structure control system consists of two parts: approaching motion towards the sliding mode surface and sliding mode motion surface along the sliding mode surface. The reachability of sliding mode only needs to reach the sliding mode transition surface from any point in the state space at a finite time, but the path it reaches is not clearly defined. Using the approach law to derive the required SMC law can enable the system to reach the sliding mode with a predetermined reaching path and speed. Therefore, the four common approach laws in Fig 1 are expressed as shown in Eq (4).


$$\{\begin{array}{c} \dot{z}=-\sigma \mathrm{sgn}(z),\sigma >0\\ \dot{z}=-\sigma \mathrm{sgn}(z)-jz,\sigma >0,j>0\\ \dot{z}=-j{\left|z\right|}^{\alpha }\mathrm{sgn}(z),j>0,0<\sigma <1\\ \dot{z}=-\sigma \mathrm{sgn}(z)-f(z),\sigma >0 \end{array}$$ (4)

In Eq (4), the first to fourth rows represent the constant velocity convergence law, exponential convergence law, power-law convergence law, and general convergence law, respectively. Among them, *σ* represents the approach speed control parameter. *j* represents the exponential approach control parameter. *α* represents the power-law approach speed control parameter.

**Fig 2:**
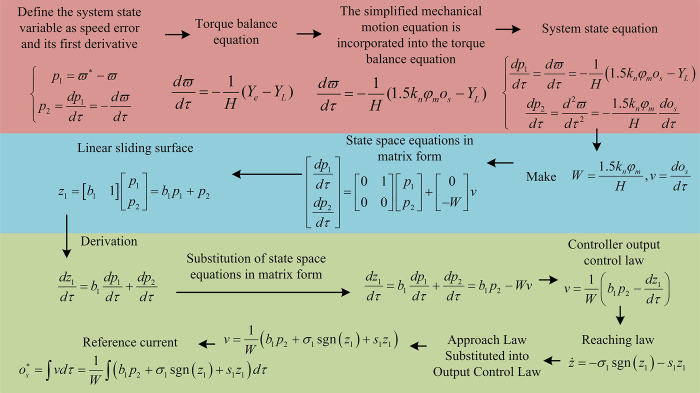
Mathematical analysis process of sliding mode speed controller design based on traditional EAL.

By analyzing four commonly used convergence methods, EAL includes both a constant velocity convergence term and an exponential convergence term. In approaching motion, EAL combines constant speed convergence and exponential convergence, with the advantage of faster approaching speed and shorter time consumption. In addition, during the synovial movement, the velocity of EAL will converge to 0. Compared with asymptotic convergence, EAL can ensure convergence in a short time, but constant speed convergence, power-law convergence, and general convergence laws cannot meet the requirements. Therefore, EAL is chosen as the PMSM speed control scheme. The mathematical analysis process of designing a sliding mode speed controller using traditional EAL is shown in Fig 2.

From Fig 2, the process first defines the system state variable as the speed error and its first derivative, and obtains the torque balance equation, mechanical motion equation, system state equation combined with the electrode model, and state space equation. Secondly, the state equation is substituted into Eq (2) to obtain a linear sliding surface expression, and its derivative is taken, while the state space expression is substituted into it. Then, the results are utilized to inverse solve the control law of the controller output, and a approach law is designed based on the EAL expression in Eq (4). Next, the designed approach law expression is substituted into the controller output control law expression to obtain the final control law expression. Finally, based on *v*, the reference current expression for the q-axis (one of the current components under the dq framework in PMSM, which is used to control the magnitude of the force, also known as the quadrature axis, which will not be elaborated on in detail in the text due to space limitations) is obtained. Among them, ϖ* represents the given speed value of PMSM, which is a constant. *ϖ* represents the actual operating speed of PMSM. *s* represents the dimension of the system output vector. $o_{s}^{*}$ represents the reference current. *H* represents the rotational inertia of PMSM. *Y*_*e*_ represents the angular velocity of PMSM mechanical rotation. *Y*_*L*_ represents the load matrix. *W* represents winding mutual inductance. *φ*_*m*_ represents the amplitude of the permanent magnet flux.

From the final expression of the control law, the corresponding control input obtained using the traditional EAL contains a discontinuous term *σ*_1_ sgn(*z*_1_), which is a conversion function and is converted from a positive and negative sign to a negative binary. The conversion amplitude depends on the size of *σ*_1_, which is the main reason for the occurrence of chattering phenomenon, and the actual chattering size also depends on the size of *σ*_1_. Therefore, when the system state is closer to the actual sliding mode surface, only the constant velocity approach term in the EAL and the time expression of the traditional EAL approach the sliding mode switching surface are considered, as shown in Eq (5).


$$\{\begin{array}{c} dz_{1}=-\sigma_{1}\mathrm{sgn}(z_{1})d\tau \\ z(0)=\int_{0}^{\tau_{1}}\sigma_{1}\mathrm{sgn}(z_{1})d\tau \end{array}$$ (5)

In Eq (5), *τ*_1_ represents the time when the traditional EAL approaches the sliding mode switching surface. The equation for the second row can be obtained by integrating the first row of Eq (6) from 0 to *τ*_1_ with the existence of the final value condition *z*(*τ*_1_) = 0.

### 3.2. PMSM speed control based on a new sliding mode EAL

In the study in section 3.1, when EAL reaches the surface of the synovial membrane, higher speeds will cause shaking. To eliminate the interdependence between arrival time and jitter level, research improved the traditional EAL and proposed a new sliding mode EAL. By introducing a sliding mode switching function, it achieved adaptive adjustment of the approximation speed, reduced the response time of the controller, and effectively suppressed flutter while ensuring the approximation speed. To further reduce chattering, a switch control function with continuous zero points was selected to replace the original intermittent switching function. The switch control function of selecting continuous zero is based on the requirement of reducing system jitter. The traditional intermittent switching function will cause severe jitter when the system state is close to the sliding surface, while the continuous zero control function can switch smoothly and reduce this jitter. This choice is based on the in-depth analysis of system dynamic characteristics and the pursuit of smooth control performance. By introducing the switch control function of continuous zero, not only the system jitter was effectively suppressed, but also the efficiency of the controller was maintained. The specific expression is shown in Eq (6).


$$\{\begin{array}{c} \overset{*}{z}=-\frac{\sigma }{E(z)}sigmoid(z)-j_{2}z^{s}\\ \frac{\sigma }{E(z)}=\gamma_{0}+(j_{1}-\gamma_{0})e^{-\alpha {\left|z\right|}^{\beta }} \end{array}$$ (6)

In Eq (6), *γ*_0_ represents a constant between 0 and 1, and *α* and *β* are positive integers. *E*(*z*) has a continuous zero switching control function. *j*_1_ is a positive integer greater than or equal to 1, *j*_2_ is greater than 0, and *s* is a positive odd number. According to Eq (7), the new AAL includes two parts: a variable speed term and an exponential approach term. When the system is far from the sliding surface, *E*(*z*) approaches *γ*_0_, so $\frac{\sigma }{E(z)}$ converges to $\frac{\sigma }{\gamma_{0}}$. At this point, its value is larger than *σ*, and the approach speed of $\frac{\sigma }{E(z)}$ increases during the approach process, reducing the time required for the system to reach the sliding surface. In addition, when the system approaches the sliding mode surface, *E*(*z*) tends to approach *j*_1_, so $\frac{\sigma }{E(z)}$ converges to $\frac{\sigma }{j_{1}}$. At this point, its value does not exceed *σ*, and $\frac{\sigma }{E(z)}$ gradually decreases during the sliding mode motion, which can limit the excessive chattering of the system. Overall, the new AAL proposed in the study theoretically adapts to changes in system state variables through dynamic changes in $\frac{\sigma }{E(z)}$, thereby achieving global fast approach of the system. It is worth noting that in Eq (7), the *sigmoid*(*z*) function is used to replace the intermittent function sgn(*z*) to reduce the chattering caused by discontinuous switching of its zero points.

Unlike traditional convergence laws, the new sliding mode asymptotic convergence law is mainly optimized in four aspects: convergence time, switching gain, discretization switching bandwidth, and controller gain. In the optimization of approach time, the expression of the new approach law and arrival time is shown in Eq (7).


$$\{\begin{array}{c} dz\cdot \frac{\gamma_{0}+(j_{1}-\gamma_{0})e^{-\alpha {\left|z\right|}^{\beta }}}{\sigma sigmoid(z)}=-d\tau \\ \tau^{\prime }=\frac{1}{\sigma }\int_{0}^{\left|z(0)\right|}\frac{\gamma_{0}+(j_{1}-\gamma_{0})e^{-\alpha {\left|z\right|}^{\beta }}}{sigmoid(z)}dz \end{array}$$ (7)

In Eq (7), *τ*′ represents the arrival time of the new AAL. Comparing the actual approach speed difference between traditional and new approach laws, assuming that the actual control parameters *σ* and *σ*_1_ in the two approach laws are equal, the time difference required for the two approach laws to approach the sliding mode switching surface is expressed in Eq (8).


$$\begin{array}{l} \Delta \tau =\tau^{\prime }-\tau_{1}=\frac{1}{\sigma }\int_{0}^{\left|z(0)\right|}\frac{\gamma_{0}+(j_{1}-\gamma_{0})e^{-\alpha {\left|z\right|}^{\beta }}}{sigmoid(z|}dz-\frac{\left|z(0)\right|}{\sigma_{1}}\\ \;=\frac{1}{\sigma }\left[\int_{0}^{\left|z(0)\right|}\frac{\gamma_{0}+(j_{1}-\gamma_{0})e^{-\alpha {\left|z\right|}^{\beta }}}{sigmoid(z)}dz-|z(0)|\right]=\frac{1}{\sigma }\int_{0}^{\left|z(0)\right|}\left[\frac{\gamma_{0}+(j_{1}-\gamma_{0})e^{-\alpha {\left|z\right|}^{\beta }}}{sigmoid(z)}-1\right]dz \end{array}$$ (8)

In Eq (8), Δ*τ* represents the difference in time required for the two approach laws to actually approach the sliding mode switching surface. The positivity and negativity of Eq (8) depend entirely on the relative size of the $\gamma_{0}+(j_{1}-\gamma_{0})e^{-\alpha {\left|z\right|}^{\beta }}$ and *sigmoid*(*z*) functions in the interval [0,|*z*(0)|]. In this case, if both are positive values, it can be considered to compare the two functions by subtracting them, as expressed in Eq (9).


$$f(z)=\gamma_{0}+(j_{1}-\gamma_{0})e^{-\alpha {\left|z\right|}^{\beta }}-sigmoid(z)$$ (9)

In Eq (9), *f*(*z*) represents the difference between two functions. From Eq (9), the difference function is a decreasing function. Therefore, when the actual control parameter *σ* and *σ*_1_ in the two approach laws are equal, the actual approach time of the new AAL is theoretically shorter than that of the traditional EAL. In the analysis of switching gain optimization, assuming that the actual approach speeds of the two approach laws are equal, i.e. assuming that *τ*′ and *τ*_1_ are equal, the expression of the existence is shown in Eq (10).


$$\frac{1}{\sigma }\int_{0}^{\left|z(0)\right|}\frac{\gamma_{0}+(j_{1}-\gamma_{0})e^{-\alpha {\left|z\right|}^{\beta }}}{sigmoid(z)}dz=\frac{\left|z(0)\right|}{\sigma_{1}}$$ (10)

According to Eq (11), it can be inferred that *σ* is less than *σ*_1_. When the approach velocities of the two approach laws are equal, the gain of the new approach law is smaller than that of the conventional EAL, thus effectively suppressing chattering. In the analysis of discretized switching bandwidth, the expression of the sliding mode switching bandwidth after discretization of the AAL and EAL is shown in Eq (11).


$$\{\begin{array}{c} \Delta =\frac{\sigma G}{j_{1}}\psi \\ \Delta_{1}=\sigma_{1}G \end{array}$$ (11)

In Eq (11), Δ and Δ_1_ represent the width of the sliding mode switching band after discretization of the AAL and the EAL, respectively. *G* represents the sampling period. *ψ* represents variables related to the system state. Comparing the two line equations, after selecting the control parameters, the switching bandwidth of the conventional EAL is an invariant non-zero value, indicating that under the influence of the EAL, the system state cannot reach the equilibrium point of the phase plane, and instead oscillates near it. However, as the state of the system approaches the sliding surface, the switching bandwidth of the new AAL approaches zero, meaning that under the action of the AAL, the system can reach an equilibrium point in the state space. Finally, a comparison is made between the two schemes in terms of controller gain. Compared with the traditional sliding mode asymptotic convergence law, when the convergence speeds of the two convergence laws are equal, the gain of the new convergence law is smaller than that of the conventional exponential convergence law. This means that under the new asymptotic approaching law, the controller’s response to system state changes is smoother, which can effectively suppress the occurrence of chattering phenomenon. In addition, by reducing the controller gain, the new asymptotic approaching law can reduce the oscillation amplitude of the system, thereby improving the stability and robustness of the system. In addition, reducing the controller gain can also reduce sensitivity to system parameter changes and external disturbances, making the system more reliable. Therefore, based on the AAL proposed in the study, the sliding mode speed controller is designed to enable the actual speed of PMSM to quickly and stably track the set speed, and to have good anti-interference performance in the presence of internal and external load disturbances in the system. Among them, the selection of state variables and basic theoretical derivation are consistent with the EAL, while the controller output obtained by using the AAL is expressed in Eq (12).


$$v=\frac{1}{W}(bp_{2}+\frac{\sigma }{\gamma_{0}+(j_{1}-\gamma_{0})e^{-\alpha {\left|z\right|}^{\beta }}}sigmoid(z)+j_{2}z^{s})$$ (12)

On the basis of Eq (12), the q-axis reference current output from the outer loop of the sliding mode speed controller using AAL is expressed as shown in Eq (13).


$$o_{qref}=\frac{1}{W}\int (b_{1}p_{2}+\frac{\sigma }{\gamma_{0}+(j_{1}-\gamma_{0})e^{-\alpha {\left|z\right|}^{\beta }}}sigmoid(z)+j_{2}z^{s})d\tau^{\prime }$$ (13)

In Eq (13), *o*_*qref*_ represents the q-axis reference current output from the outer loop of the sliding mode speed controller using AAL. In addition, to prove the high stability of the system under the action of the new sliding mode AAL proposed in the study, the Lyapunov scalar function is also selected as *U* = *z*^2^/2, and the expression of the first-order derivative of the Lyapunov scalar function is shown in Eq (14).


$$\dot{U}=z\dot{z}=z(-\frac{\sigma }{\gamma_{0}+(j_{1}-\gamma_{0})e^{-\alpha {\left|z\right|}^{\beta }}}sigmoid(z)-j_{2}z^{s})$$ (14)

In Eq (14), since *s* is a positive odd number, $\dot{U}$ is less than or equal to 0, which means that the AAL can ensure that the trajectory of the system’s state motion eventually converges to the switching surface. When it reaches the switching surface, the corresponding equation is expressed as shown in Eq (15).


$$z=bp_{1}+p_{2}=bp_{1}+{\dot{p}}_{1}=0$$ (15)

**Fig 3:**
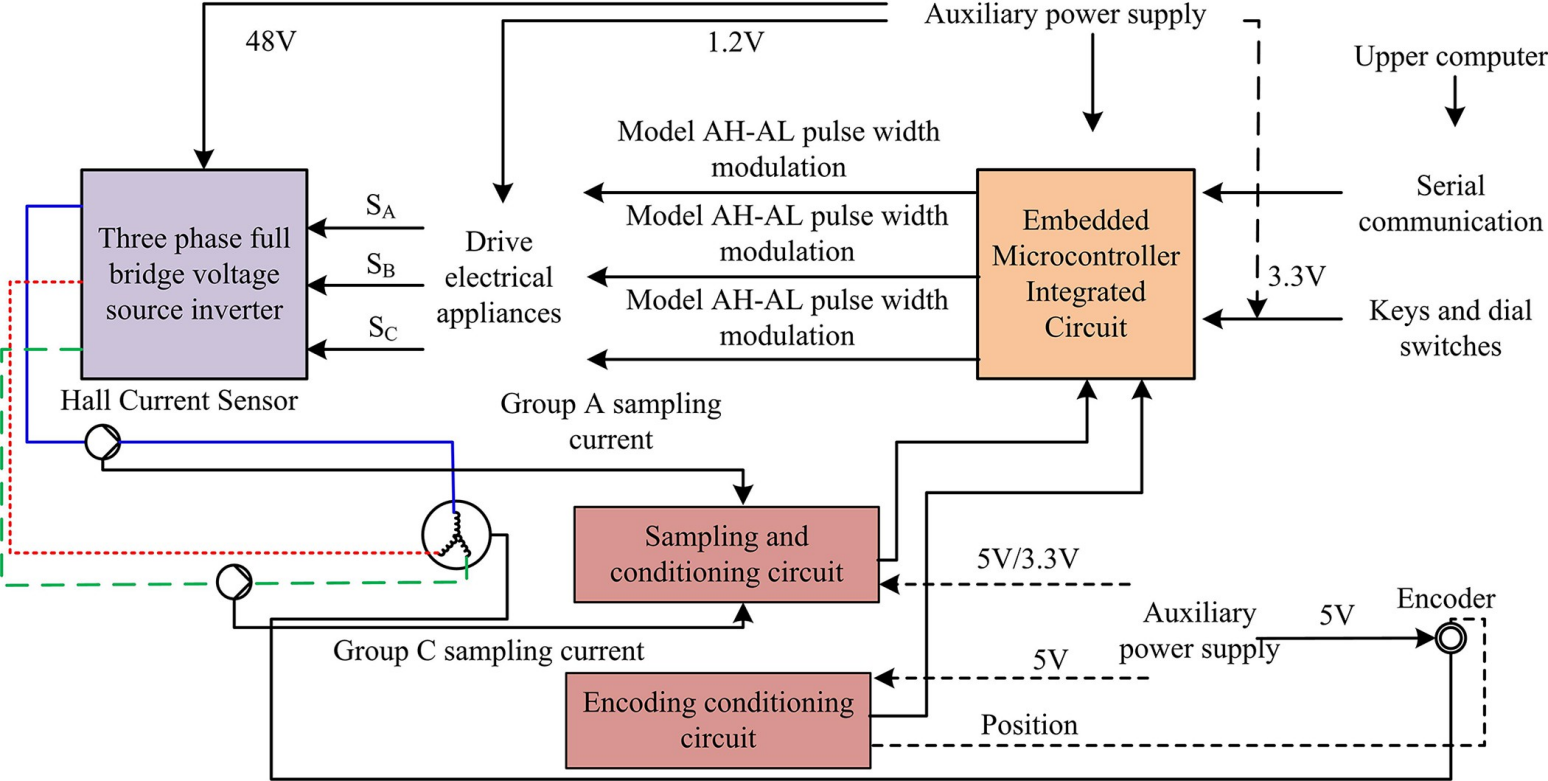
Hardware structure diagram of PMSM speed control system driver board.

From Eq (15), the actual speed error of the system will eventually converge to 0, which means that the system can achieve speed tracking without overshoot. In theory, the system can achieve stability under the proposed AAL algorithm. In the subsequent experimental analysis, the research will set the speed control system using PID algorithm as PID, the speed control system using traditional EALSMC as SMC, and the speed control system using new AALSMC as ASMC. The hardware structure of the PMSM speed control system driver board selected in the experimental verification of the ASMC algorithm is shown in Fig 3.

From Fig 3, the hardware circuit of the PMSM speed control system mainly includes auxiliary power supply circuit, main circuit, sampling and conditioning circuit, interface circuit, etc. The focus is on the sampling and conditioning circuit. Although the node voltage is directly provided by the regulated power supply module in the sampling and conditioning circuit, in practical applications, the power switch will constantly switch, which leads to fluctuations in this signal. Therefore, a first-order resistor capacitor filter was chosen for filtering to solve this problem. Subsequent actual experiments were conducted in this hardware architecture.

## 4. PMSM speed control simulation experiment

### 4.1. ASMC system performance simulation analysis

**Fig 4:**
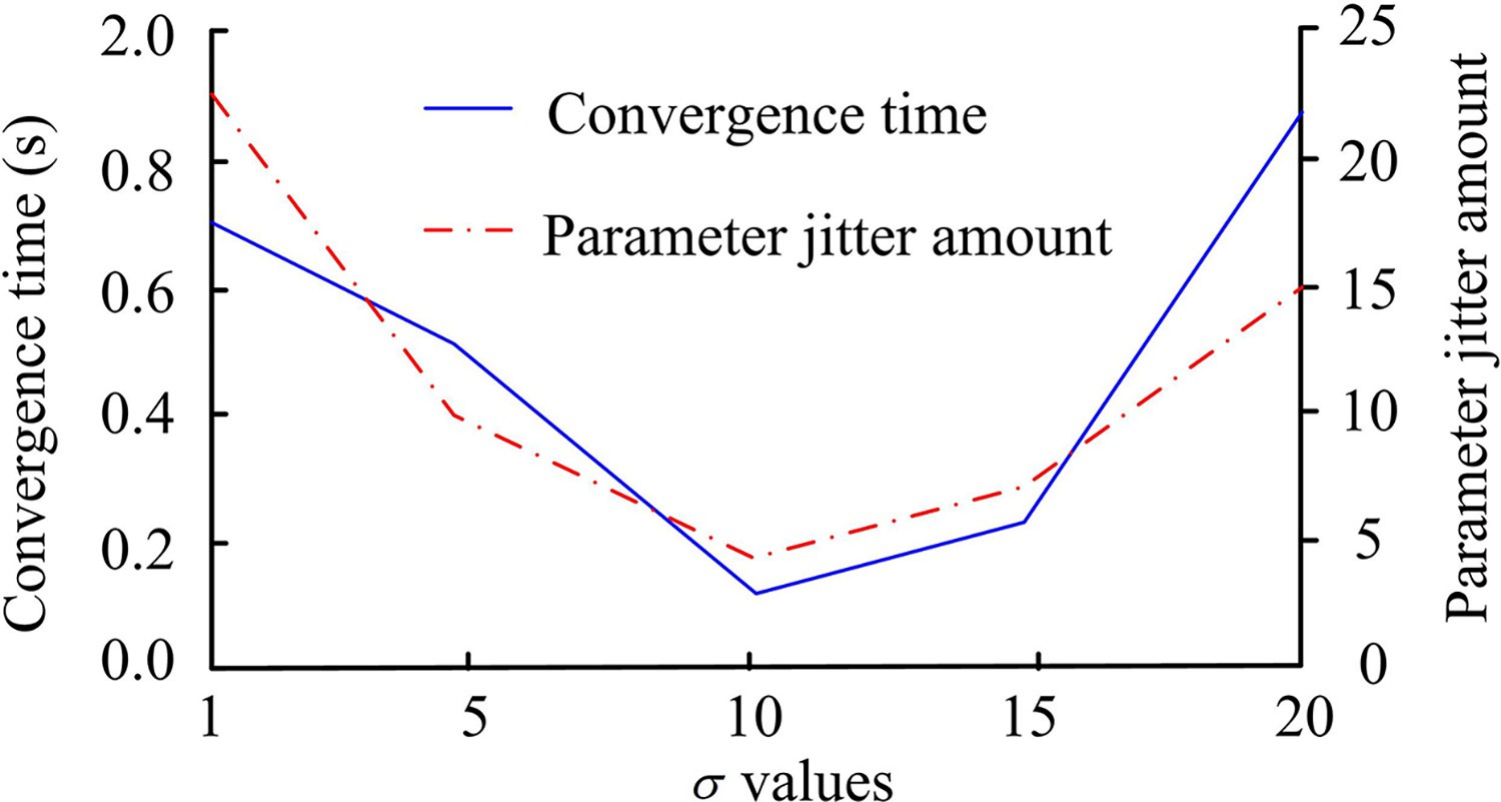
Sensitivity analysis of ASMC *σ* parameter.

To better describe the influence of different parameters on the performance of the proposed method, the ASMC algorithm was be systematically simulated in the experiment. Before the experiment, set the control *σ* in the convergence law parameters for comparison, with parameters set to 1, 5, 10, 15, and 20 respectively. The remaining parameters are based on the default parameters, Both *α* and *β* are set to 2, Set *j*_1_ and *j*_2_ as default values in ASMC control, which is 10. The sensitivity analysis of parameter *σ* is shown in Fig 4.

**Fig 5:**
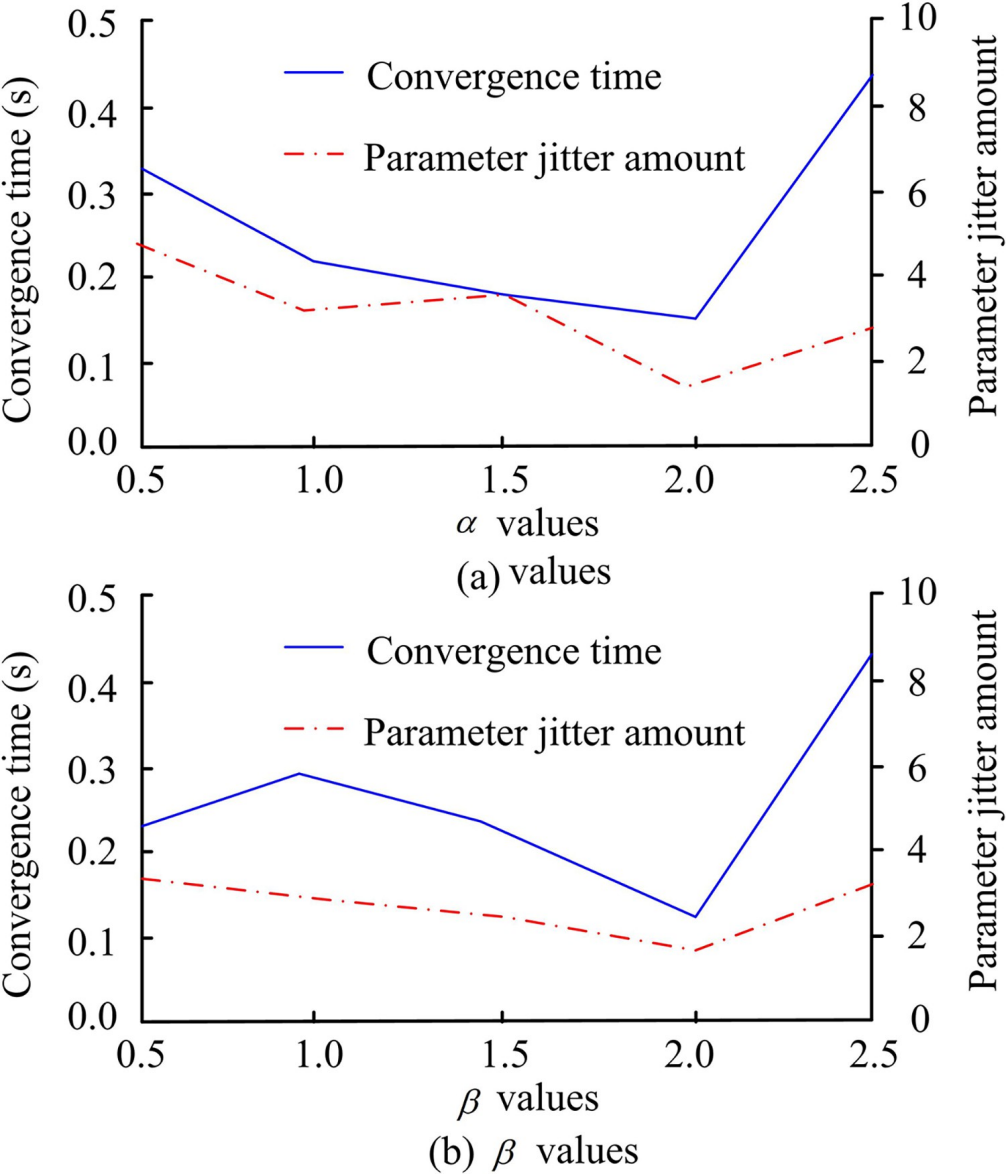
Sensitivity analysis of parameters *α* and *β*.

According to the results in Fig 4, the convergence of ASMC is not the same under different parameters. When the parameter *σ* is 10, the shortest convergence time is 0.192s, and the minimum flutter value is 5.12. Therefore, the *σ*-value was set to 10 in the experiment. Next, the sensitivity analysis results of parameters *α* and *β* are shown in Fig 5.

**Fig 6:**
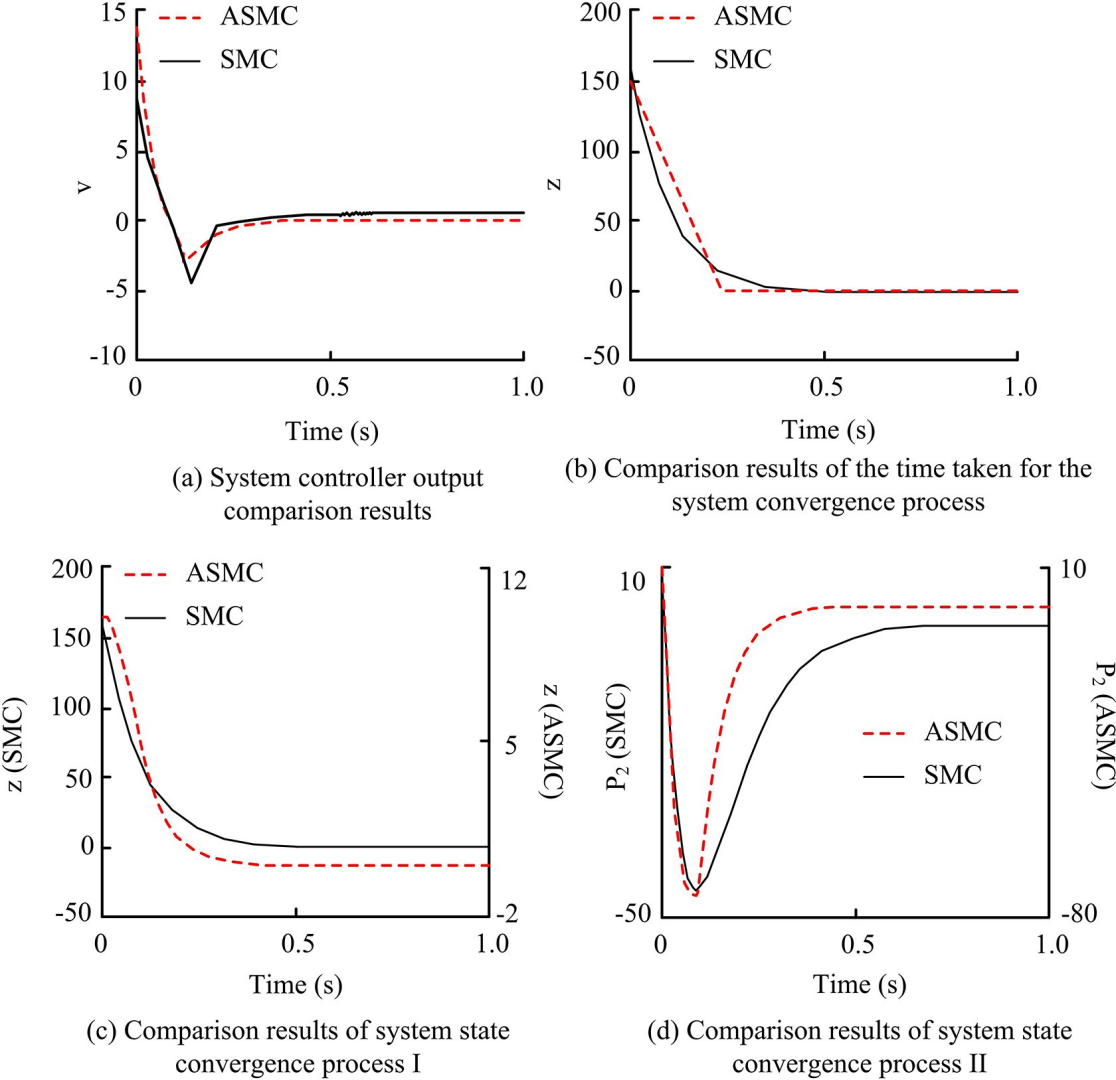
Comparison results of the performance of traditional approach law control and new AAL control.

Among them, Fig 5(A) shows the test results of parameter *α*, and Fig 5(B) shows the values of parameter *β*. The shortest convergence time for *α* is 0.812s, with the shortest time and the smallest jitter. Meanwhile, when *β* is 2, the convergence time is also the shortest and the jitter is the smallest. Therefore, in the experiment, r is set to 10, Both *α* and *β* are set to 2. Next, the comparison results of the performance between the traditional convergence law control and the new asymptotic convergence law control are shown in Fig 6.

**Fig 7:**
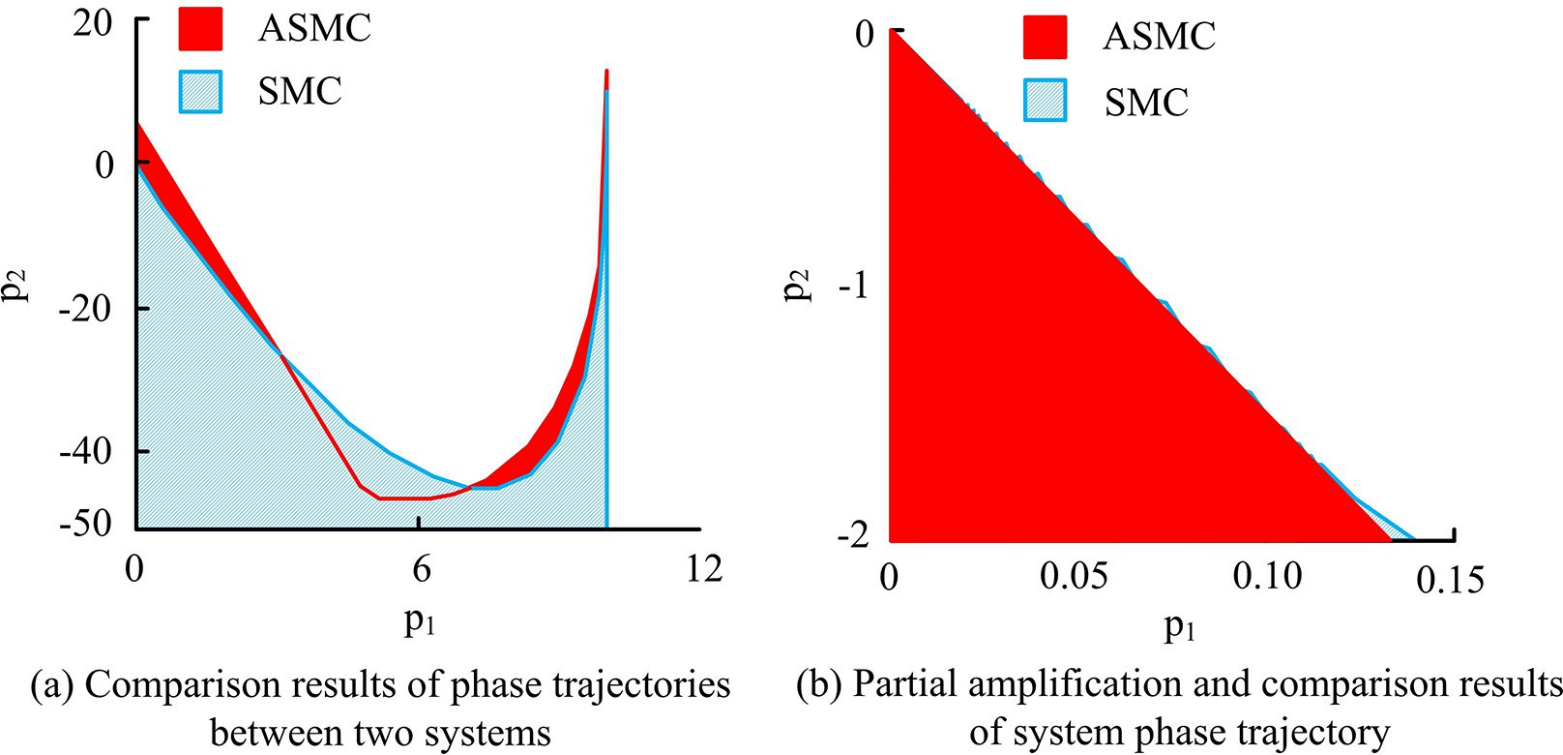
Comparison results of phase trajectories and local enlarged images between two systems.

From Fig 6(A), it can be seen that the system control requires parameter chattering to be controlled within ± 4. Under the same parameters, the ASMC system chattering is within the range of ± 3, and the ASMC system tends to stabilize within 0.2–0.3 seconds, while the SMC system tends to stabilize within 0.6–0.7 seconds, with parameter chattering exceeding the range of ±3. For more obvious vibration problems, shock-absorbing decorations can be installed, such as shock-absorbing springs or adjusting the center of gravity of the rotor, to reduce the vibration phenomenon. In Fig 6(B), the SMC system takes about 0.5 seconds to achieve sliding surface convergence, while the ASMC system takes about 0.1 seconds to achieve sliding surface convergence. Therefore, it can be seen that the ASMC system achieves convergence faster. In the comparison between the convergence state process I and process II, the convergence time of the ASMC system is significantly shorter. For example, in the system convergence process I, it can be seen that the ASMC converges to the 0 position, while the SMC converges to the 11.5 position, indicating that the ASMC system has lower chattering and better suppression effect. To see the results more clearly, the phase trajectory and local enlarged image were analyzed, and the results are shown in Fig 7.

**Fig 8:**
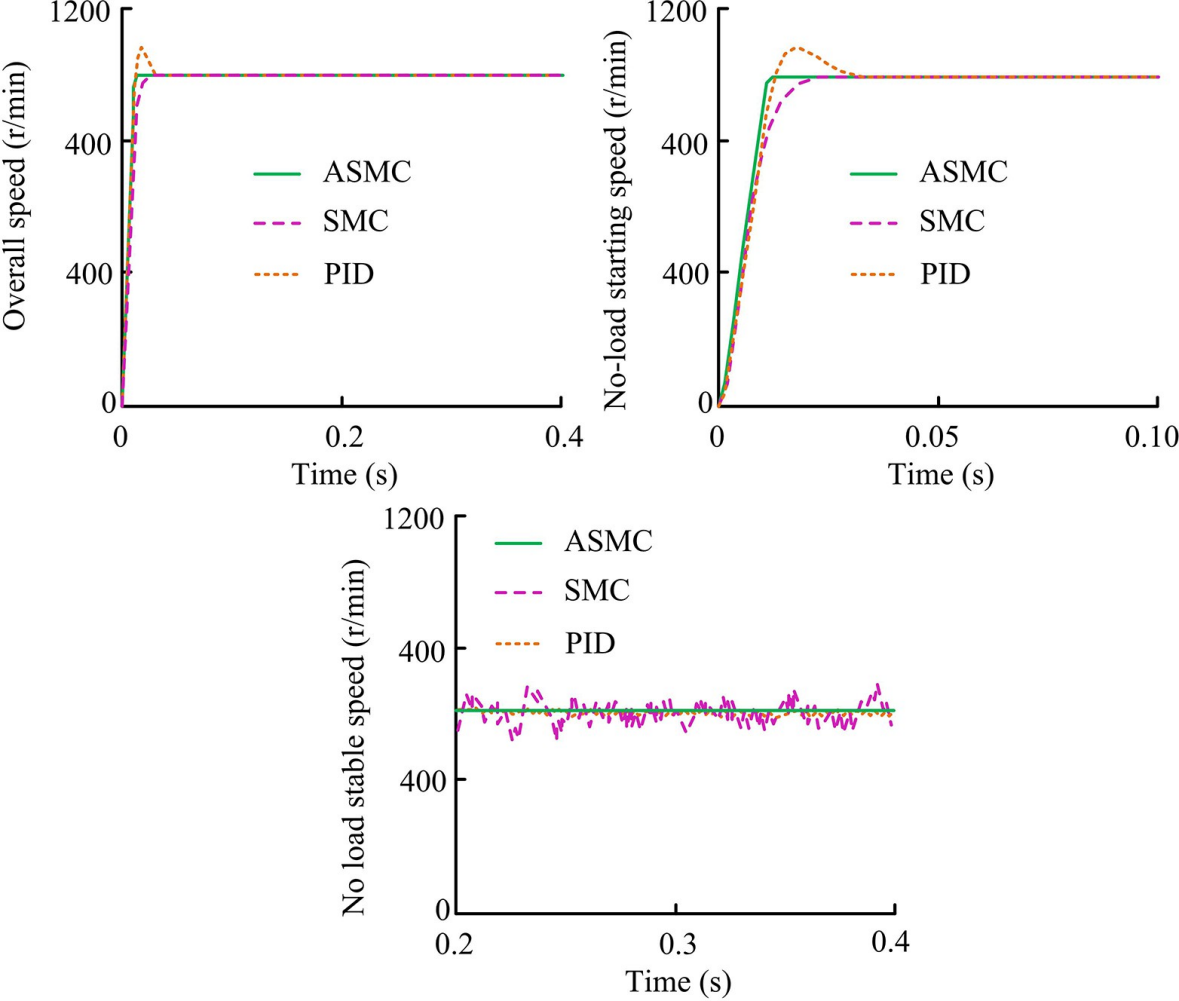
Comparison results of three system related speeds.

From Fig 7, the actual chattering level of the SMC system was significantly higher, with more severe chattering at *p*_1_ between 0.05 and 0.10. In the ASMC system, its chattering was significantly and effectively weakened. Overall, the AAL could significantly improve the performance indicators of buffet level, approach time, and phase trajectory waveform compared to the EAL, improving the dynamic motion quality of the system. On this basis, it researched and analyzed the relevant indicators of PID, SMC, and the ASMC system proposed in the study under the condition of no disturbance (which refers to the condition that the motor’s body parameters remain unchanged and the external load torque is constant zero during operation). The comparison outcomes of the three system related speeds are denoted in Fig 8.

**Fig 9:**
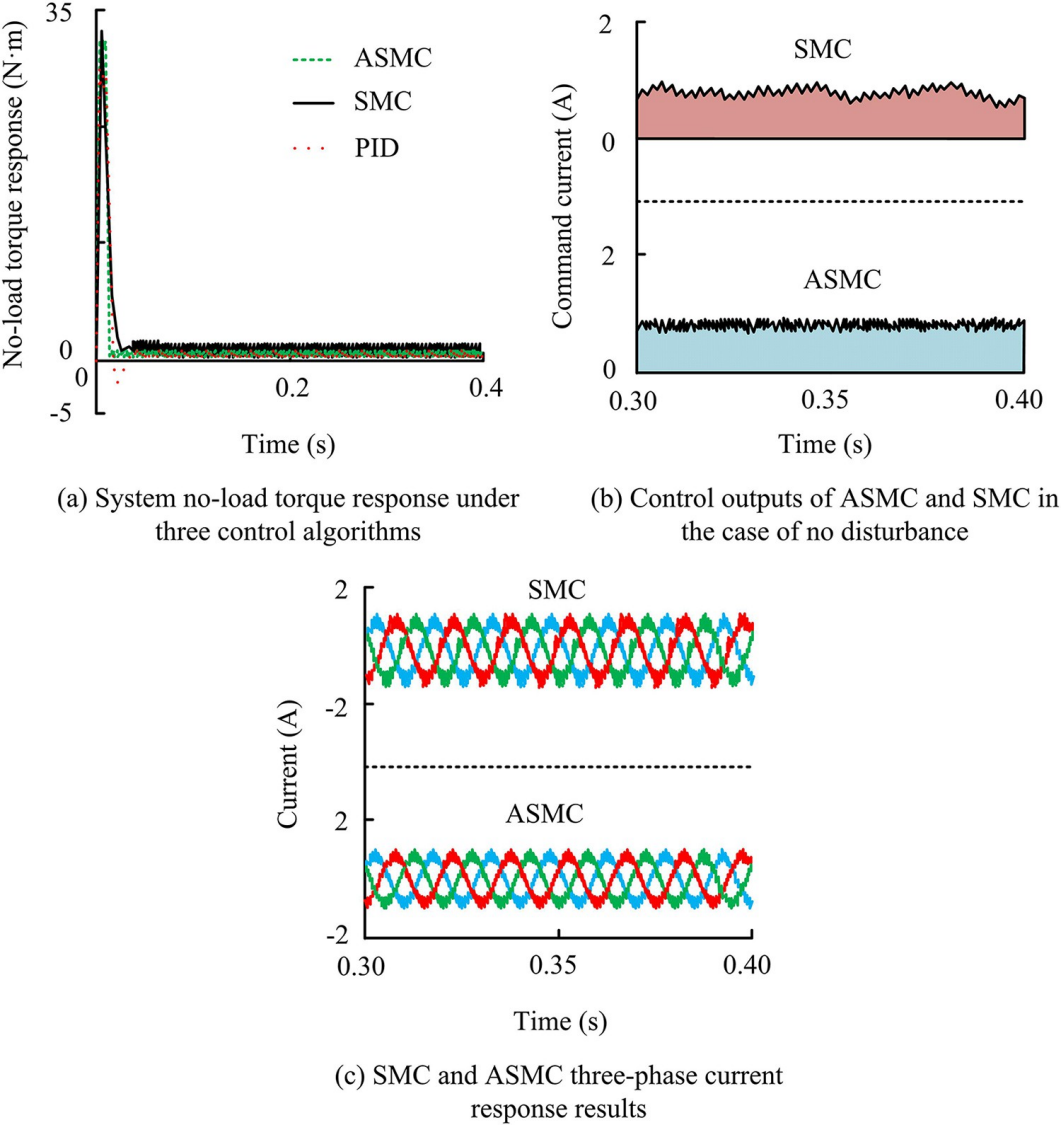
System no-load torque response results under three control algorithms.

From Fig 8, under the actual action of the three controllers, the response speed of PMSM could track the given actual command speed without static error. During the PMSM startup and operation process, the actual adjustment time of the ASMC system was the shortest, about 0.013 seconds, and there was no overshoot during the overall startup phase. Although the SMC system also did not experience overshoot, its actual adjustment time was 0.037 seconds, exceeding the ASMC system by 0.024 seconds. The PID system would experience an overshoot of approximately 8.39%, with an actual adjustment time of 0.047 seconds and a maximum speed of 1084 r/min. Overall, the ASMC system had the optimal startup dynamic performance, and when the PMSM was under stable operating conditions, the ASMC system’s chattering was significantly reduced compared to the SMC system. This further confirmed the performance of the AAL in suppressing chattering. The system no-load torque responseunder the three control algorithms, control output of ASMC and SMC without disturbance, and three-phase current response results are shown in Fig 9.

In Fig 9, the speed in the no-load torque response analysis was set to 1000r/min, and the simulation time was set to 0.4s. From Fig 9, the torque fluctuation when the system reached steady state was very small, while the SMC system showed significant fluctuations, with an amplitude of approximately 1.2 N·m, indicating that using the AAL could make the torque response of the system more stable, thereby making the motor work more stable. In addition, after entering the steady state, both PID and ASMC had the same torque response. However, before entering the steady state, PID control would experience significant negative overshoot, making it take longer to reach the steady state. However, the ASMC system was always greater than 0 and at a positive value, indicating that its speed was better than PID. Meanwhile, compared to the SMC system, the output of the ASMC control system was smoother and the jitter amplitude was significantly reduced by 1/3, which helped to achieve accurate tracking of the actual current and indirectly made the output torque of the motor more stable. Overall, the stability of the ASMC system was stronger, indicating that the AAL was effective.

**Fig 10:**
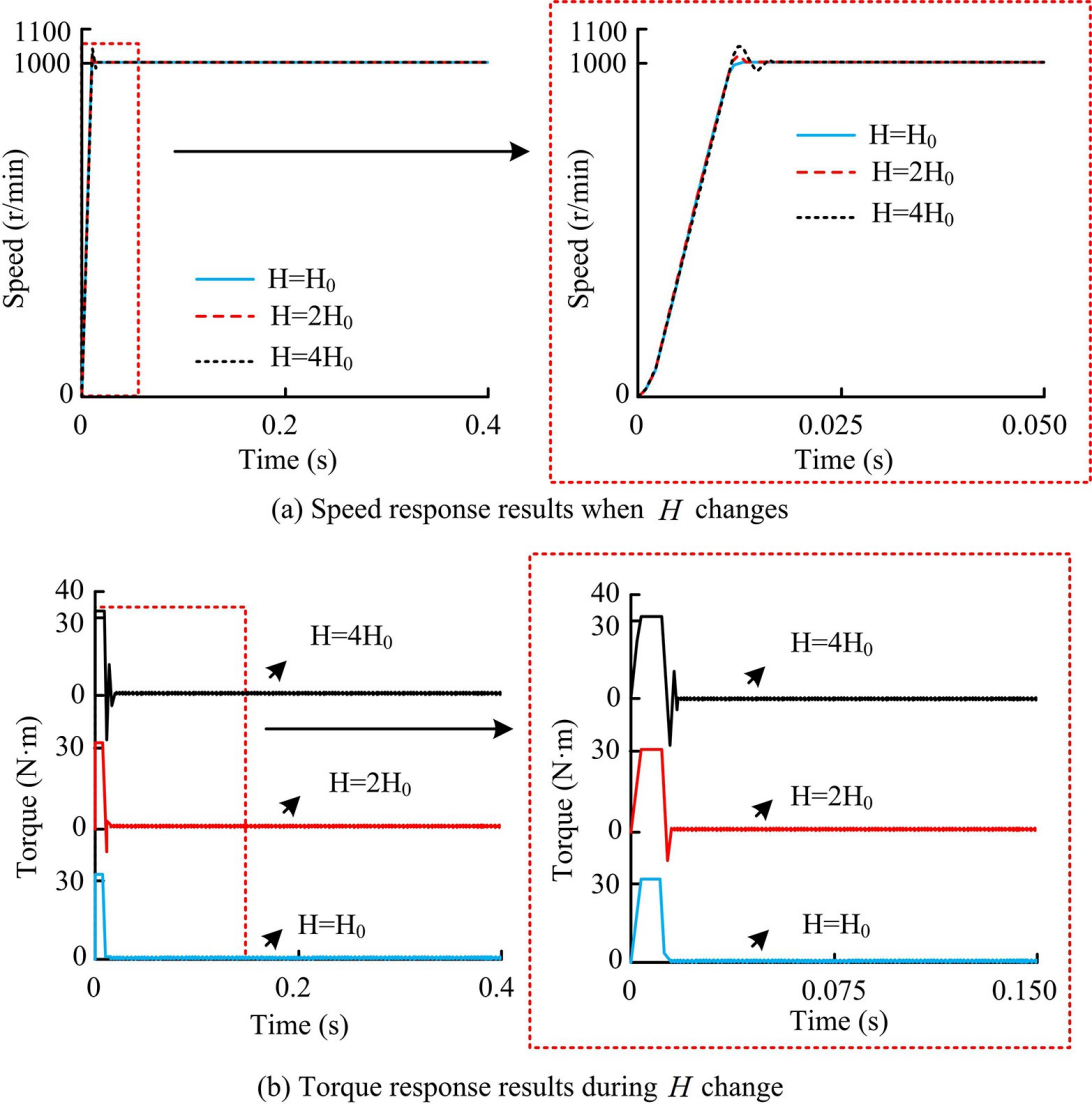
Speed response and torque response results when parameter *H*_0_ changes.

In practical systems, it is difficult to achieve accurate results solely by measuring the inertia moment and other parameters of the motor. Therefore, research analyzed the corresponding situation of the system under the perturbation of motor parameters. Among them, the sliding mode speed control system was derived from the mechanical motion equation of the system, so it had a dependency on the moment of inertia parameter *H*. Therefore, research was conducted to verify the robustness of the ASMC system to *H* changes based on different changes in parameter *H*. In the experiment, the initial moment of inertia of the motor was set to *H*_0_, so the speed response and torque response results during parameter *H* perturbation are shown in Fig 10.

**Fig 11:**
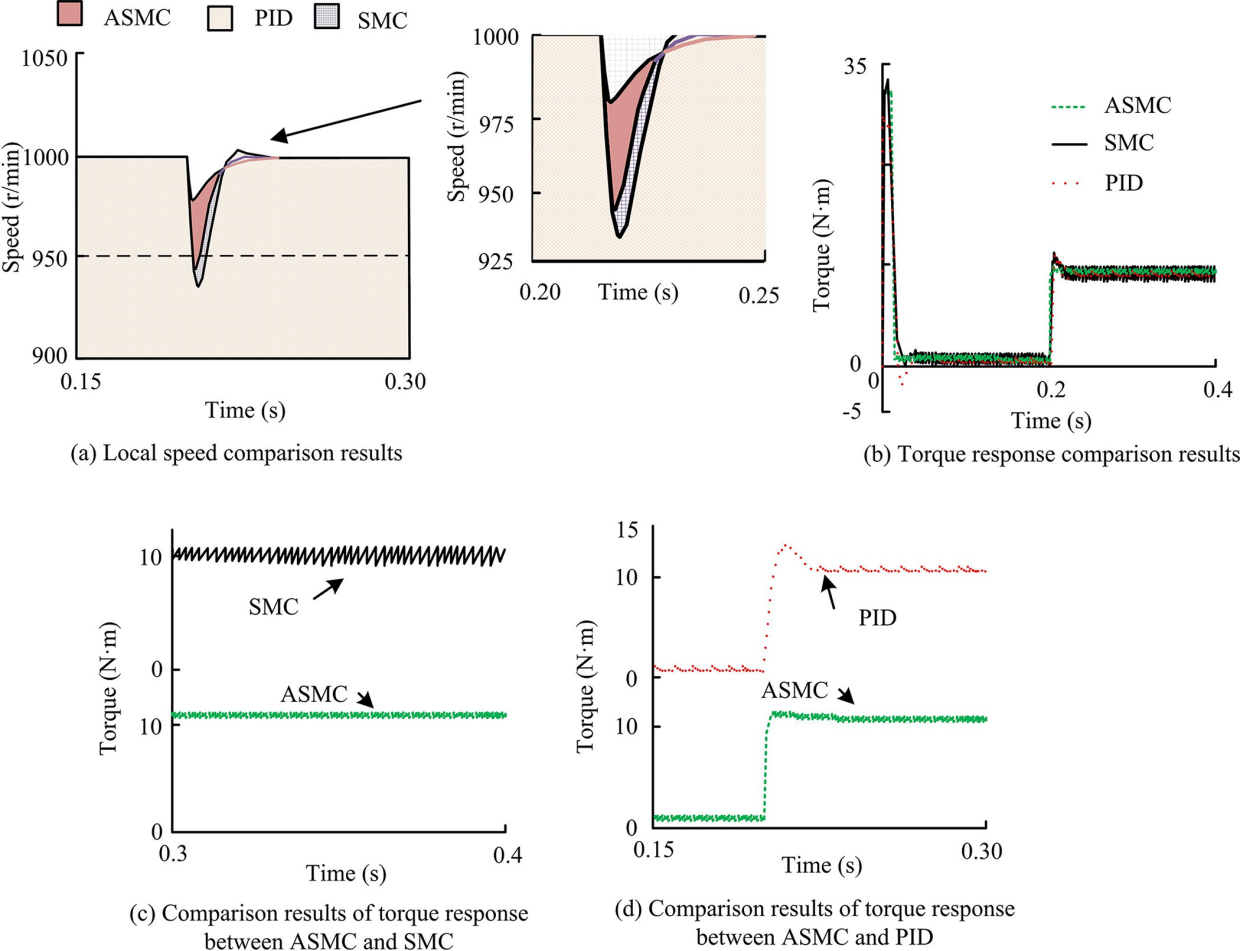
Simulation results of sudden reduction of load torque.

From Fig 10, in the limited rotational inertia domain, overall, the actual speed of the motor could still keep up with the commanded speed and maintain consistency. The total output torque response of the electric motor was also the same, but there were significant differences in some places. Between 0.01s and 0.05s, when the parameter *H*_0_ changed towards 2*H*_0_, the overall speed curve did not change much, with only a slight overshoot. The overshoot was only 2.8%, and the change amplitude remained around 25r/min. The torque curve adjustment time would be slightly extended, and the time it took to enter steady state would be slightly increased. When the parameter *H*_0_ changed to 4*H*_0_, there would be a significant change in the speed curve, with an overshoot of 4.7% and a change amplitude of around 50r/min. Moreover, the adjustment time would also be extended, and the control performance of the system would be more affected by inertia. Overall, ASMC could adapt to the moment of inertia within a certain range and had good self stability performance. On this basis, research was conducted to add external load disturbances in the simulation to verify the anti load disturbance ability of ASMC, which included two situations: sudden torque increase and decrease. Among them, in the simulation of sudden increase in load torque, the initial value of load torque was set to 0, the step time was 0.2s, the step value was 10N·m, and the simulation time was 0.4s. The results are shown in Fig 11.

**Fig 12:**
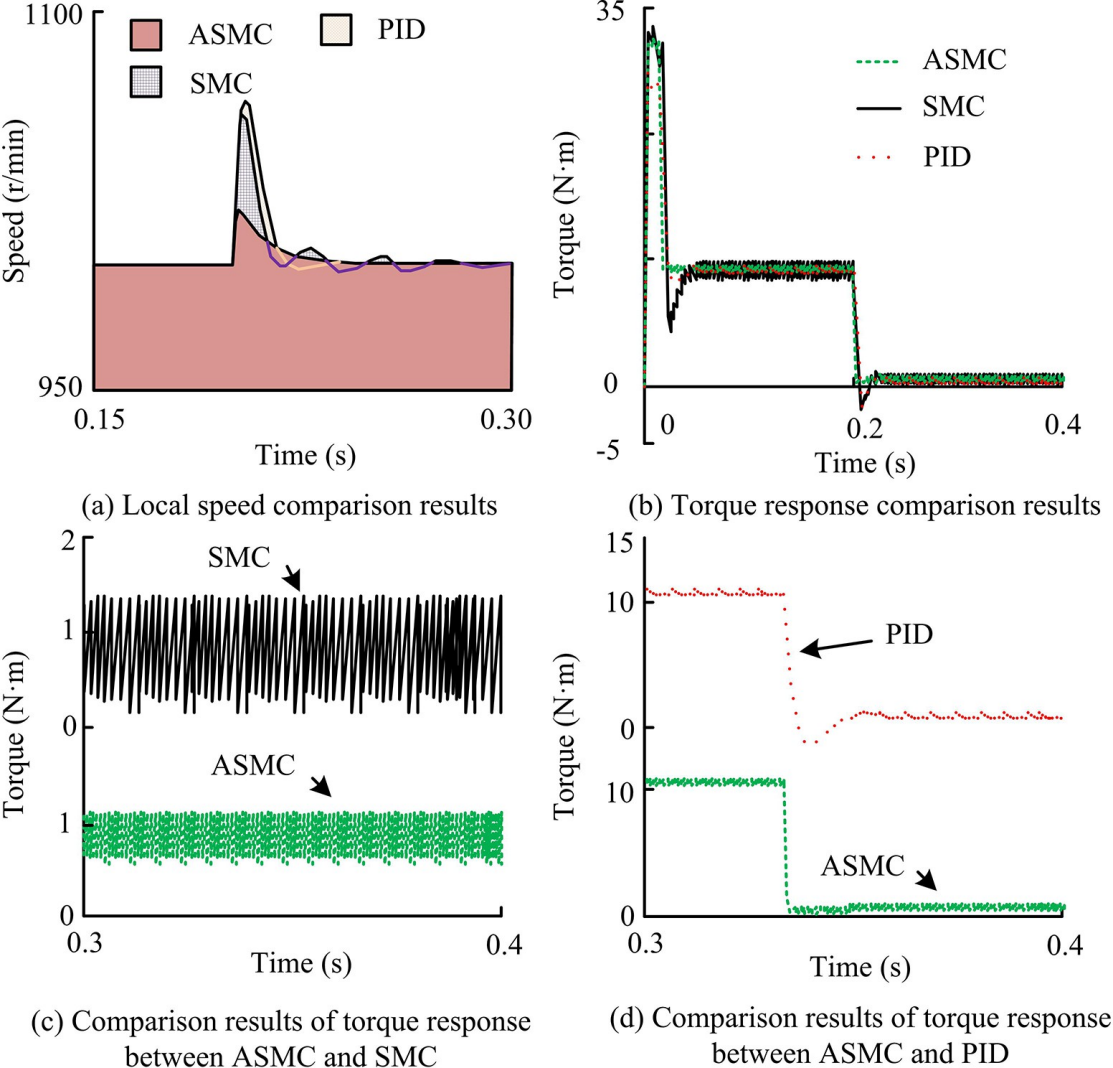
Simulation results of sudden reduction of load torque.

From Fig 11, under external load disturbances, all three systems had varying degrees of speed reduction, and they all needed to undergo some adjustment before returning to the original level. But the ASMC system had the smallest speed decrease, only around 21r/min. After adjusting for 0.03s, it quickly returned to the required speed and continued to operate steadily. The speed of PID control decreased to the maximum of 64r/min, and the dynamic adjustment speed was slow. The adjustment time of the three parameters was the maximum of 0.06s. Among the three systems, the SMC system’s deceleration ratio and power adjustment time were in the middle position. In addition, the output torque of all three systems was maintained at around 10N·m, which was near the load torque, indicating that all three systems could maintain good working conditions. However, after steady-state, the torque ripple of the SMC system was much larger than that of the ASMC and PID systems. The local graph further confirms that the ASMC system has significantly reduced chattering. When the load torque suddenly decreases by 0.22, only the ASMC system’s local speed chattering fluctuates within ± 50r/min, which meets the requirement of chattering fluctuation within ±50r/min under load interference. However, traditional PID control has obvious vibration problems, requiring additional damping systems, such as adding damping blocks or pads to reduce control vibration and ensure the effective operation of motor equipment. In addition, when the three systems encounter sudden load reduction, only the ASMC system can recover stability within 0.02 seconds, while the PID system and SMC system need 0.03 to return to stability, indicating that the ASMC system has better resistance to load disturbances. In the simulation of sudden reduction of load torque, the initial value of load torque was set to 10N. m, the step time was 0.2s, the step value was 0, and the simulation time was 0.4s. The results are shown in Fig 12.

From Fig 12 the sudden decrease in load torque was a completely symmetrical process compared to the sudden increase, so the simulation results were basically the same as the results of the sudden increase. Based on Figs 9 and 10, compared with traditional AMC systems, the ASMC system significantly improved its chattering level, and also significantly improved its dynamic response and robustness to load disturbances compared to the PID system.

### 4.2. Practical application of ASMC in PMSM speed control system

To evidence the correctness of ASMC, the study applied it to the actual PMSM speed control system. The motor drive control circuit is shown in Fig 3, and the relevant parameters of PMSM and magnetic powder brake are expressed in Table 2.

**Table 2:** Parameters related to PMSM and magnetic particle brakes.

Electrical parameters of the motor					
-	Rated voltage	Rated current	Rated power	Rated speed	Rated torque
Numerical value	60.00V	8.40A	400.00W	3000.00r/min	1.27N·m
-	Peak torque	Moment	Number of pole pairs	Rotational inertia	Permanent magnet flux chain
Numerical value	3.82N·m	0.16 (N·m)/A	4.00	0.09g·cm2	0.020Wb
Mechanical parameters of the motor					
-	Frame size	Shaft Diameter	Output shaft length	Total length of motor	-
Numerical value	60.00mm	8.40mm	400.00mm	3000.00mm	
Magnetic particle brake parameters					
-	Model	Rated torque	Rated current	Rated power	Maximum speed
	Baogoma ZKB0S6	6.00N·m	0.81A	19.40W	1800.00r/min

**Fig 13:**
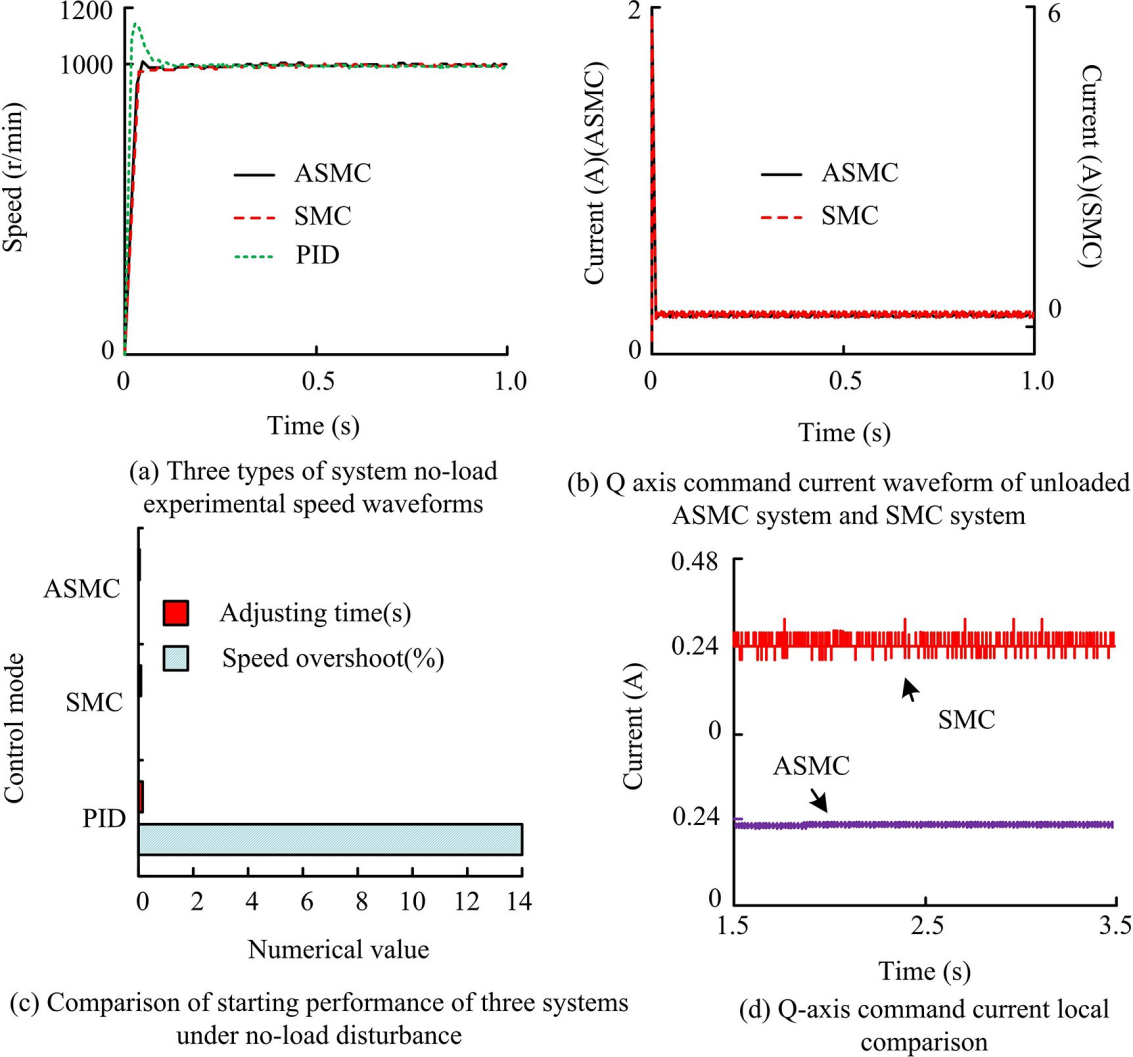
No-load experimental speed waveform and Q-axis command current waveform of ASMC and SMC systems compared with local results.

From Table 2, the rated voltage, current, power, speed, and torque in the electrical parameters of the motor were 60V, 8.4A, 400W, 3000r/min, and 1.27N·m, respectively. In the mechanical parameters of the motor, the base size and shaft diameter were 60mm and 8.4mm respectively, and the maximum speed of the magnetic powder brake reached 1800r/min. In this practical application system, performance verification was conducted for three systems under three operating conditions: disturbance free, parameter perturbation, and load disturbance. Among them, under the condition of no disturbance, the reference speed was set to 1000r/min. Under the action of three controllers, the motor was respectively started from no load to stable operation state, that is, under the condition of global no disturbance, and the experimental time was kept the same, the no-load experimental speed waveforms of the three systems, as well as the q-axis command current waveforms of the no-load ASMC and SMC systems and the local comparison results are shown in Fig 13.

From Fig 13, the actual speed of the PID system under undisturbed no-load conditions had a certain degree of overshoot, with a maximum no-load speed of 1140r/min and an overshoot of 14%. By comparing the adjustment time of these three control methods, the PID control callback had the maximum lag value of 0.15s, while the adjustment time difference between SMC and ASMC control systems was not significant, with 0.09s and 0.04s, respectively. In addition, compared with the SMC system, the current command oscillation of the ASMC system was significantly reduced. The local graph showed that the output fluctuation amplitude of the AAL actual control was significantly smaller in the absence of load disturbance, indicating that the system oscillation was effectively weakened. Overall, the ASMC system had the fastest response among the three control methods, tracked the instruction speed earliest and operated smoothly, and the AAL effectively suppressed system chattering. This result was consistent with simulation experiments, indicating the performance of ASMC.

**Fig 14:**
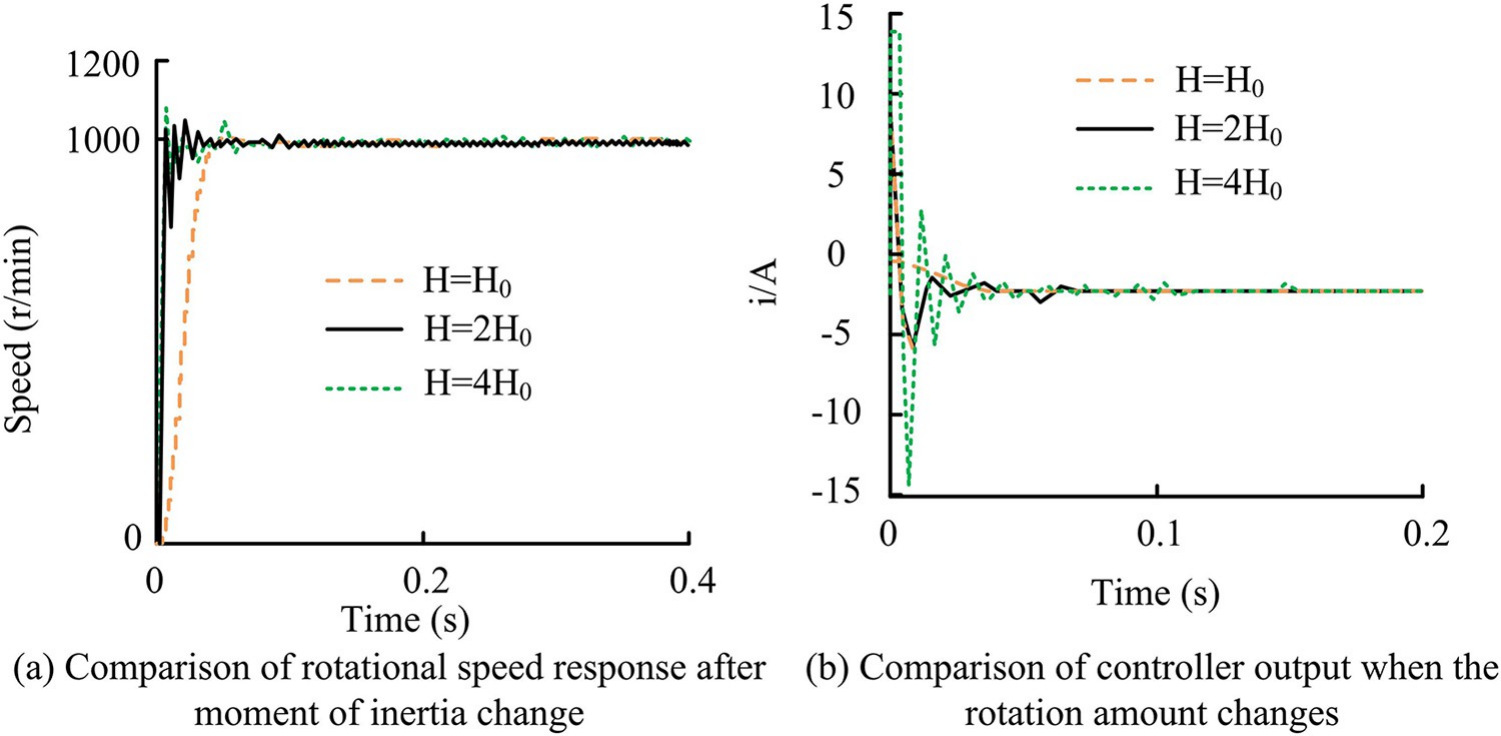
Speed response curve and torque response curve after the change of moment of inertia.

According to the different changes of moment of inertia, the simulation analysis of ASMC system was carried out to verify the robustness of ASMC system to H change. In the system experiment under the condition of parameter perturbation, the reference speed was set to 1000r/min, and the whole process was not loaded. Only by changing the lower computer program, the moment of inertia parameters changed in a certain range. The speed response curve and torque response curve after the change of moment of inertia are shown in Fig 14.

**Fig 15:**
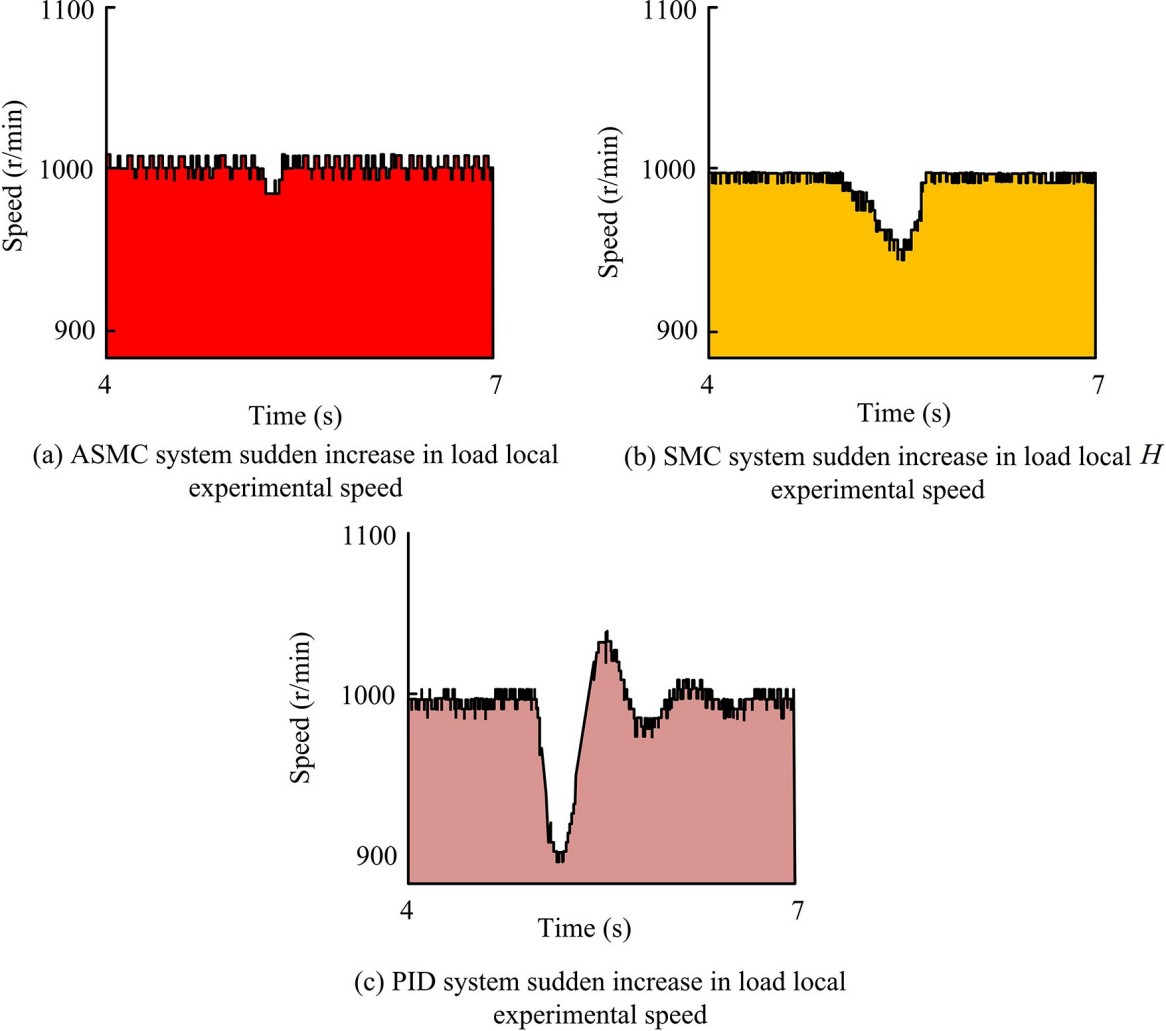
Partial experimental speed waveform results under sudden load increase of three systems.

From Fig 14(A), when H0 changed to 2H0, overshoot occurred, and the overshoot rate was 5%. At this time, the speed curve changed little. However, when 2H0 changed to 4H0, there was also overshoot, and the overshoot rate was 8%. At this time, the speed curve changed significantly, and the adjustment time would become longer, and the time for the torque response to enter the steady state would be obviously lengthened. From Fig 14(B), when 2H0 changed to 4H0, the control performance of the system was significantly affected by the change of moment of inertia. When the moment of inertia changed within a certain range, the actual speed of the motor could track the given speed instruction as a whole, but the response of the speed would be different. Generally speaking, ASMC system hadgood adaptability within a certain range, but beyond this range, it would have an impact on the control effect of the system. This was basically consistent with the results of simulation experiments, which provedthe practicability of ASMC, that is, the new AAL proposed by the study was practical. At the same time, in the system experiment under load disturbance, the reference speed was set to 1000r/min, the magnetic particle brake current was fixed at 0.3A, and the load input time was uniformly set to 5s. That is, the motor was first started with no load and entered a stable operating state, and then the power supply of the magnetic particle brake was suddenly turned on at the 5-th second to simulate the sudden load application condition. Therefore, the local experimental speed waveform results under sudden load increase of the three systems are shown in Fig 15.

**Fig 16:**
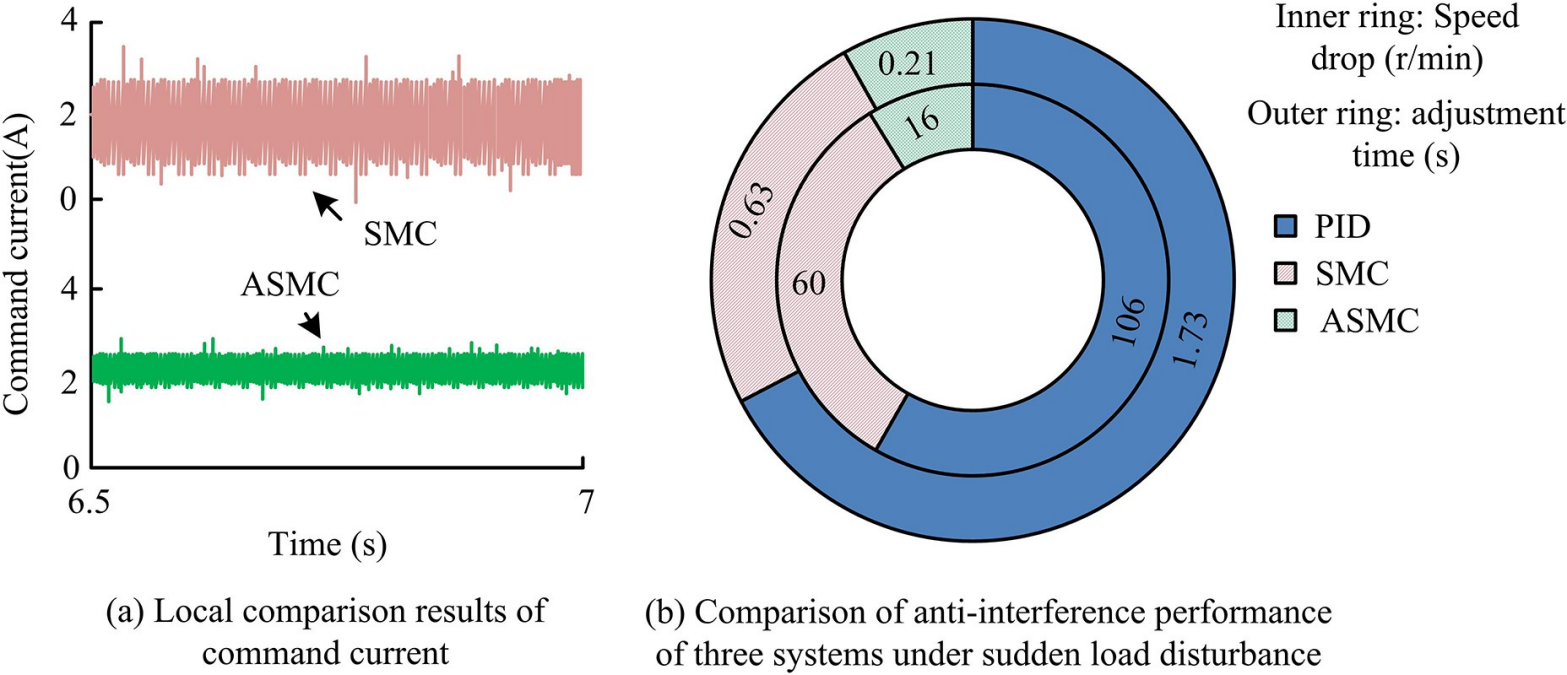
Comparison results of local Q-axis command current of ASMC and SMC systems under sudden load increase and anti-interference of three systems.

From comprehensive Fig 15, when external load disturbance occurred, the speed of PID system dropped most significantly, with the maximum value of 106r/min, while after load disturbance, the adjustment time was about 1.73 seconds. The speed decrease amplitude controlled by SMC could reach 60r/min, and the adjustment time could reach 0.63s. The speed decrease amplitude and adjustment time were significantly better than those of PID controllers. The speed decrease rate using the ASMC system was only 16r/min, and the adjustment time was also only 0.21s. Overall, the ASMC system had the lowest actual deceleration and the shortest adjustment time, and its dynamic characteristics were the best, with the strongest resistance to load disturbances. Finally, the study analyzed the local comparison of the q-axis command current between ASMC and SMC systems under sudden load increase, as well as the comparison of the anti-interference performance of the three systems, as shown in Fig 16.

From Fig 16, the fluctuation of the ASMC system was significantly lower than that of the SMC system, and the fluctuation amplitude was significantly reduced, about one-third of the original, indicating that it effectively suppressed system chattering. In addition, in the comparison of the anti-interference performance of the three systems under sudden load disturbance, the PID speed drop value was 106r/min and the adjustment time was 1.73s. The SMC speed drop value was 60r/min, and the adjustment time was 0.63s. The ASMC speed drop value was 16r/min, and the adjustment time was 0.21s. Overall, the AAL proposed in the study significantly suppressed system chattering and improved the speed control quality of the system, which was consistent with simulation experiments and further proved the practicality of ASMC.

To compare the startup performance of the proposed ASMC control strategy more intuitively, the overshoot and adjustment time were selected as comparison indexes and compared with other existing methods. Other existing methods were the maximum net power strategy of sliding mode variable structure control system based on exponential approach law in reference [20] and the adaptive second-order SMC strategy based on back stepping method in reference [21]. The comparison results of the three strategies are shown in Table 3.

**Table 3:** Comparison of startup performance of three strategies under the same load disturbance.

Method type	Speed overregulation of (%)	accommodation time (s)
ASMC control strategy	1.28	0.05
Maximum net power strategy of	6.31	0.11
Adaptive second-order sliding mode control strategy	11.35	0.16

From Table 3, under the same load disturbance, there were some overshoots in the ASMC control strategy, the maximum net power strategy and the adaptive second-order SMC strategy, and the speed overshoots were 1.28%, 6.31%, and 11.35% respectively. The adjustment time of the three strategies was 0.05s, 0.11s, and 0.16s respectively. The proposed ASMC control strategy had the fastest response among the three, tracking the commanded speed first and running stably. Finally, in a real experimental environment, the output power of the synchronous motor can be changed by changing the excitation current, achieving load adjustment in the drag test. Meanwhile, an adaptive integral sliding mode predictive control (AISMPC) technique from reference 18 was introduced for comparison, and the results are shown in Table 4.

**Table 4:** Comprehensive performance effects of three control systems under sudden load and sudden load reduction conditions.

Indicator items	PID	SMC	ASMC	AISMPC
Maximum speed after startup (r/min)	1246	1221	1096	1120
Overshoot after startup	0.345	0.212	0.096	0.201
Peak time after startup/s	0.0452	0.0374	0.0166	0.0301
Minimum speed after sudden load increase/(t/min)	965	975	998	979
Overregulation after sudden load application	0.023	0.021	0.005	0.017
Stable time after sudden load application/s	0.416	0.435	0.421	0.439
Maximum speed after sudden load reduction/(t/min)	1075	1069	1020	1051
Stable time after sudden load reduction/s	0.244	0.256	0.261	0.243
Overregulation after sudden load reduction	0.058	0.065	0.025	0.51

**Fig 17:**
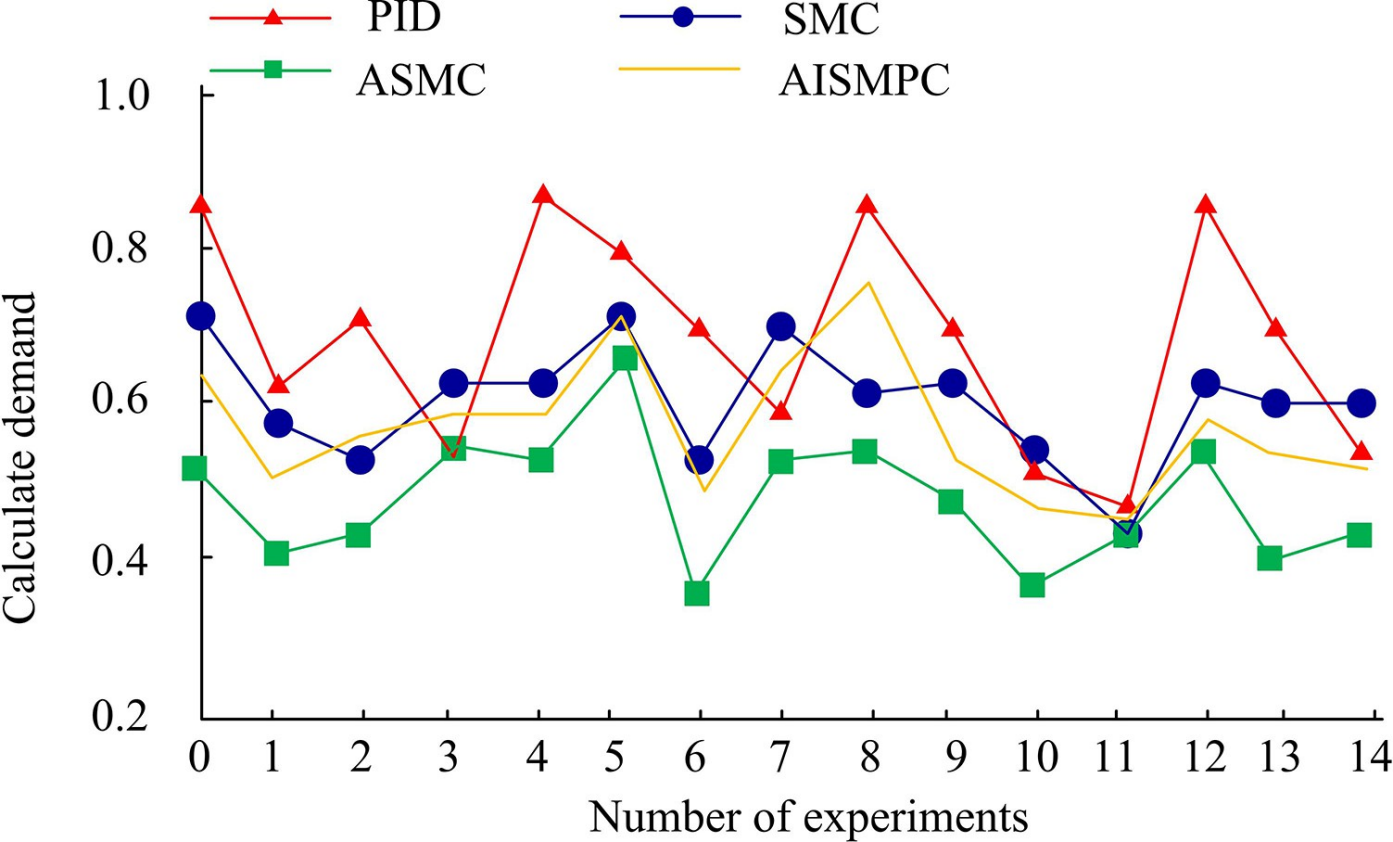
Comparison of computational requirements for different control systems.

Table 4 shows the comprehensive performance comparison results of two control systems. Select PID A comprehensive comparison of four control systems: SMC, ASMC, and Reference 18. Firstly, compare the three highest system speeds when the system starts in an instant state, PID, SMC, ASMC, and Reference 18 have speeds of 1246r/min, 1221r/min, 1096r/min, and 1120r/min, respectively, The ASMC system has the lowest speed during startup, and the control effect at the highest speed is the best among the three, with a minimum overshoot of 0.096. In the control of sudden load, The minimum speeds for PID, SMC, ASMC, and AISMPC are 965r/min, 975r/min, 998r/min, and 979r/min, respectively, The ASMC speed is closer to 1000r/min, and the control effect is better. It can be seen that the ASMC system has a longer stable control time and better control effect. In addition, the calculation requirements of ASMC were compared, as shown in Fig 17.

According to the results in Fig 17 and 14 different scenario strategy demand rate comparisons were conducted, such as experiments 0, 4, 8, 12, etc., which were high load control scenarios. In high load scenarios, the traditional PID calculation requirements exceed the system calculation requirements by 80%, and the system requires a large amount of resources to participate in the calculation. The best performing ASMC has the lowest computational demand in all 14 scenarios, and the highest computational demand in high load scenarios is 0.69, indicating that ASMC has good application effects in real-world scenarios.

## 5. Conclusion

The PID control of traditional PMSM in industrial manufacturing can no longer meet the requirements of high-performance industrial control. Therefore, in order to improve the speed control effect of PMSM, a high-performance sliding mode variable structure control algorithm ASMC is proposed based on AAL research, and its performance is verified. In the convergence of system control, The ASMC system tends to stabilize within 0.2–0.3 seconds, The SMC system requires 0.6s to 0.7s, and the shaking is more severe. In response speed control analysis, ASMC can control speed in the shortest time of 0.013, while SCM PID requires 0.037s and 0.047s respectively, and overshoot phenomenon has occurred. In the analysis of PMSM control system, PID overshoot occurred under undisturbed no-load conditions, with a maximum no-load speed of 1140r/min and a lag of 0.15s. However, the ASMC system performed the best with no overshoot and a control adjustment time of 0.04s. In load control, The ASMC speed control time is 0.21s and the speed decrease is stable, while the PID speed control time is 1.73s, showing a significant decrease in speed. The SMC speed control time is 0.63s, indicating a significant decrease in speed. Finally, in the analysis of sudden increase and decrease of load, The maximum starting speed of ASMC is 1096r/min, with the most stable control, and the speed control time is the shortest under sudden increase or decrease of load. It can be seen that the ASMC control system proposed by the research institute performs the best in practice. However, in practical work environments, the load often changes over time. In future research, it can be considered to combine a load observer that can accurately observe time-varying disturbances to improve the adaptability and practicality of the ASMC method in practical speed control systems, and guide future research and development work in this field.

## Supporting information

S1 Dataset Minimal data set definition. (DOCX)
