# Supplementary material for: Application of sliding mode variable structure control algorithm in PMSM vector control system in complex environment
Source: PLoS One. 2024 Sep 13;19(9):e0308417. doi: 10.1371/journal.pone.0308417 (PMC11398649; doi:10.1371/journal.pone.0308417)
Supplement: S1 Dataset — (DOCX) [file pone.0308417.s001.docx]

The minimum dataset content has been added in the final section of the paper, as shown below:

Figure 4 Dataset Content:

| Indicator type | Method type | Minimum value | Adjusting time （s） |
| --- | --- | --- | --- |
| System controller output com parison results | SMC | -4.91 | 0.31 |
|  | ASMC | -3.95 | 0.72 |
| Comparison results of the time taken for the system convergence process | SMC | 0 | 0.41 |
|  | ASMC | 0 | 0.24 |
| Comparison results of system state convergence process I | SMC | 11.5 | 0.42 |
|  | ASMC | 0 | 0.41 |
| Comparison results of system state convergence process II | SMC | -47.3 | 0.62 |
|  | ASMC | -71.1 | 0.21 |

Figure 5 Dataset Content

| Indicator type | Method type | P1 | P2 |
| --- | --- | --- | --- |
| Comparison of trajectories between two systems | SMC | 0.06 | 3.2 |
|  | ASMC | 0.06 | -1.2 |

Figure 6 Dataset Content

| Indicator type | Method type | Adjustment time (s) | Overshoot (%) |
| --- | --- | --- | --- |
| Overall speed | PID | 0.047 | 8.39 |
|  | SMC | 0.037 | 0 |
|  | ASMC | 0.024 | 0 |
| No-load starting speed | PID | 0.027 | 8.45 |
|  | SMC | 0.021 | 0 |
|  | ASMC | 0.013 | 0 |
| No load stable speed | PID | - | 4.32 |
|  | SMC | - | 0 |
|  | ASMC | - | 0 |

Figure 7 Dataset Content

Figure 7 (a)

| Indicator type | Method type | Torque ripple (N · m) | Adjustment time (s) |
| --- | --- | --- | --- |
| No-load torque response | PID | 0.9 | 0.028 |
|  | SMC | 1.2 | 0.012 |
|  | ASMC | 0.1 | 0.005 |

Figure 7 (b)

| Indicator type | Method type | Maximum amplitude of current jitter (A) |
| --- | --- | --- |
| Control Outputs of ASMC and SMC in the Case of No Disturbance | SMC | 0.092 |
|  | ASMC | 0.063 |

Figure 7 (c)

| Indicator type | Method type | Current jitter amplitude (A) |
| --- | --- | --- |
| SMC and ASMC three-phase current response results | SMC | -0.09 to 0.09 |
|  | ASMC | -0.06 to 0.06 |

Figure 8 Dataset Content

Figure 8 (a)

| Indicator type | Index | Overshoot (%) | Change amplitude (r/min) |
| --- | --- | --- | --- |
| Speed response results when *H*changess | H=H0 | 0 | 0 |
|  | H=2H0 | 2.8 | 25 |
|  | H=4H0 | 4.7 | 50 |

Figure 8 (b)

| Indicator type | Index | Maximum torque overshoot (N · m) |
| --- | --- | --- |
| Torque response results during *H*change | H=H0 | 0 |
|  | H=2H0 | 15.5 |
|  | H=4H0 | 29.6 |

Figure 9 Dataset Content

Figure 9 (a)

| Indicator type | Method type | Speed reduction (r/min) | Adjustment time (s) |
| --- | --- | --- | --- |
| Local speed comparison results | PID | 64 | 0.06 |
|  | SMC | 45 | 0.05 |
|  | ASMC | 21 | 0.03 |

Figure 9 (b)

| Indicator type | Method type | Maximum torque overshoot (N · m) |
| --- | --- | --- |
| Torque response comparison results | PID | 10 |
|  | SMC | 10 |
|  | ASMC | 10 |

Figure 9 (c)

| Indicator type | Method type | Torque ripple (N · m) |
| --- | --- | --- |
| Comparison results of torque response between ASMC and SMC | SMC | 0.5 |
|  | ASMC | 0.2 |

Figure 9 (d)

| Indicator type | Method type | System stability time (s) |
| --- | --- | --- |
| Comparison results of torque response between ASMC and PID | PID | 0.03 |
|  | ASMC | 0.02 |

Figure 10 Dataset Content

Figure 10 (a)

| Indicator type | Method type | Speed increase (r/min) | Adjustment time (s) |
| --- | --- | --- | --- |
| Local speed comparison results | PID | 58 | 0.06 |
|  | SMC | 43 | 0.05 |
|  | ASMC | 20 | 0.03 |

Figure 10 (b)

| Indicator type | Method type | Maximum torque overshoot (N · m) |
| --- | --- | --- |
| Torque response comparison results | PID | 10 |
|  | SMC | 10 |
|  | ASMC | 10 |

Figure 10 (c)

| Indicator type | Method type | Torque ripple (N · m) |
| --- | --- | --- |
| Comparison results of torque response between ASMC and SMC | SMC | 0.5 |
|  | ASMC | 0.2 |

Figure 10 (d)

| Indicator type | Method type | System stability time (s) |
| --- | --- | --- |
| Comparison results of torque response between ASMC and PID | PID | 0.04 |
|  | ASMC | 0.01 |

Figure 11 Dataset Content

Figure 11 (a)

| Indicator type | Method type | Maximum overshoot (r/min) | Adjustment time (s) |
| --- | --- | --- | --- |
| Three types of system no-load experimental speed waveforms | PID | 1140 | 0.15 |
|  | SMC | 1025 | 0.09 |
|  | ASMC | 1012 | 0.04 |

Figure 11 (b)

| Indicator type | Method type | Maximum current fluctuation (A) |
| --- | --- | --- |
| Q axis command current waveform of unloaded ASMC system and SMC system | SMC | 0.08 |
|  | ASMC | 0.01 |

Figure 11 (c)

| Indicator type | Method type | Speed overshoot(%) |
| --- | --- | --- |
| Comparison of Starting Performance of Three Systems under No Load Disturbance | PID | 14.02% |
|  | SMC | 0.02% |
|  | ASMC | 0.00% |

Figure 11 (d)

| Indicator type | Method type | Maximum current fluctuation (A) |
| --- | --- | --- |
| Q-axis command current local comparison | SMC | 0.12 |
|  | ASMC | 0.01 |

Figure 12 Dataset Content

Figure 12 (a)

| Indicator type | Index | Overshoot (%) | Adjustment time (s) |
| --- | --- | --- | --- |
| Comparison of actual speed when changes | H=H0 | 0 | 0 |
|  | H=2H0 | 5 | 0.10 |
|  | H=4H0 | 13 | 0.16 |

Figure 12 (b)

| Indicator type | Index | Maximum overshoot (r/min) |
| --- | --- | --- |
| Comparison of controller outputs when*H* changes | H=H0 | 0 |
|  | H=2H0 | 235 |
|  | H=4H0 | 723 |

Figure 13 Dataset Content

| Indicator type | Method type | Descent speed (r/min) | Adjustment time (s) |
| --- | --- | --- | --- |
| System sudden increase in load local experimental speed | Method type | 106 | 1.73 |
|  | SMC | 60 | 0.63 |
|  | ASMC | 16 | 0.21 |

Figure 14 Dataset Content

Figure 14 (a)

| Indicator type | Method type | Maximum current fluctuation (A) |
| --- | --- | --- |
| Local comparison results of command current | SMC | 1.12 |
|  | ASMC | 0.42 |

Figure 14 (b)

| Indicator type | Method type | Inner ring: Speed drop (r/min) | Outer ring: adjustment time (s) |
| --- | --- | --- | --- |
| Comparison of anti-interference performance of three systems under sudden load disturbance | PID | 16 | 0.21 |
|  | SMC | 60 | 0.63 |
|  | ASMC | 106 | 1.73 |

Table 3 Data

| Method type | Speed overregulation of (%) | accommodation time (s) |
| --- | --- | --- |
| ASMC control strategy | 1.28 | 0.05 |
| Maximum net power strategy of | 6.31 | 0.11 |
| Adaptive second-order sliding mode control strategy | 11.35 | 0.16 |

Table 4 Data

| Indicator items | PID | SMC | ASMC |
| --- | --- | --- | --- |
| Maximum speed after startup (r/min) | 1246 | 1221 | 1096 |
| Overshoot after startup | 0.345 | 0.212 | 0.096 |
| Peak time after startup/s | 0.0452 | 0.0374 | 0.0166 |
| Minimum speed after sudden load increase/(t/min) | 965 | 975 | 998 |
| Overregulation after sudden load application | 0.023 | 0.021 | 0.005 |
| Stable time after sudden load application/s | 0.416 | 0.435 | 0.458 |
| Maximum speed after sudden load reduction/(t/min) | 1075 | 1069 | 1020 |
| Stable time after sudden load reduction/s | 0.244 | 0.256 | 0.261 |
| Overregulation after sudden load reduction | 0.058 | 0.065 | 0.025 |
